# Probing Isoform Switching Events in Various Cancer Types: Lessons From Pan-Cancer Studies

**DOI:** 10.3389/fmolb.2021.726902

**Published:** 2021-11-23

**Authors:** Tülay Karakulak, Holger Moch, Christian von Mering, Abdullah Kahraman

**Affiliations:** ^1^ Department of Molecular Life Sciences, University of Zurich, Zurich, Switzerland; ^2^ Department of Pathology and Molecular Pathology, University Hospital Zurich, Zurich, Switzerland; ^3^ Swiss Informatics Institute, Swiss Institute of Bioinformatics, Lausanne, Switzerland; ^4^ Faculty of Medicine, University of Zurich, Zurich, Switzerland

**Keywords:** alternative splicing, isoform switching, pan-cancer analysis, bioinformatics tools and databases, differential transcript usage

## Abstract

Alternative splicing is an essential regulatory mechanism for gene expression in mammalian cells contributing to protein, cellular, and species diversity. In cancer, alternative splicing is frequently disturbed, leading to changes in the expression of alternatively spliced protein isoforms. Advances in sequencing technologies and analysis methods led to new insights into the extent and functional impact of disturbed alternative splicing events. In this review, we give a brief overview of the molecular mechanisms driving alternative splicing, highlight the function of alternative splicing in healthy tissues and describe how alternative splicing is disrupted in cancer. We summarize current available computational tools for analyzing differential transcript usage, isoform switching events, and the pathogenic impact of cancer-specific splicing events. Finally, the strategies of three recent pan-cancer studies on isoform switching events are compared. Their methodological similarities and discrepancies are highlighted and lessons learned from the comparison are listed. We hope that our assessment will lead to new and more robust methods for cancer-specific transcript detection and help to produce more accurate functional impact predictions of isoform switching events.

## Introduction

**FIGURE 1:**
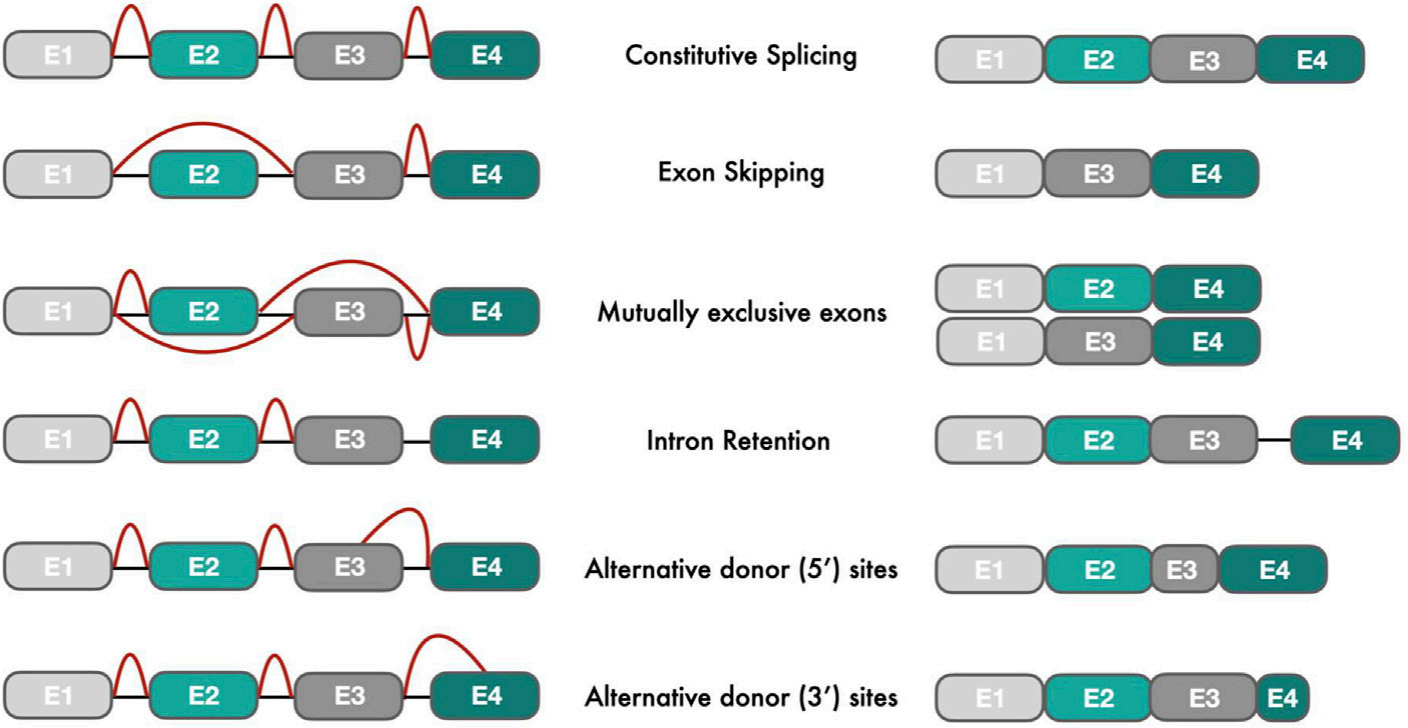
Types of alternative splicing events: The left figure shows different pre-mRNA transcripts with exons (E) in different colors and introns as black connecting lines. Red lines indicate splicing events that join exons intro processed mRNA transcripts (right part of the figure).

Alternative splicing of precursor messenger RNA (pre-mRNA) is a key regulator of gene expression in mammalian cells, causing the rearrangement of intron and exon elements into multiple RNA transcripts via the differential use of splice sites (Matera and Wang, 2014). The enzymatic reactions of alternative splicing are performed by the spliceosome, a large ribonucleoprotein complex consisting of small nuclear RNAs (snRNAs) and small nuclear ribonucleoproteins (snRNPs) (Wahl et al., 2009). The splicing mechanism proposed by Gilbert (Gilbert, 1978) changed the notation of “one gene → one RNA → one protein” to “one gene → multiple RNA transcripts → multiple protein isoforms with various functions”. On average, eight exons code for four or more isoforms per gene creating ∼86,700 protein isoforms from ∼19,700 protein-coding genes in humans (Ensembl, version 104). More than 95% of human genes with multiple exons undergo alternative splicing (Nilsen and Graveley, 2010) compared to 25% of protein-coding genes in nematodes (Ramani et al., 2011), suggesting a role of alternative splicing in the formation of organism complexity. Alternative splicing events can be classified into five major types according to splice site selection: cassette exon (exon skipping), mutually exclusive exons, intron retention, alternative 5’splice site, alternative 3’splice site (Figure 1). In addition, other pre-mRNA processing events such as alternative start, termination and promoter sites have also been described (Reyes and Huber, 2018). In higher eukaryotes, intron retention events are more frequently observed than exon skipping events (Grau-Bové et al., 2018). However, evolutionary analyses have shown that exon skipping events were the preferred mechanism in early animals for frame-preserving (frame length divisible by three) isoform generation (Grau-Bové et al., 2018). In cancer, exon skipping events are also enriched, occurring 30% more often than in normal tissues (Kahles et al., 2018).

For splicing to work properly, three main consensus splice sites must work together in the pre-mRNA: 5′ splice site, 3′ splice site, and an adenosine nucleotide at the so-called branch point, which is located 18–40 nucleotide upstream of 3′ splice site (Kelemen et al., 2013). An additional regulatory region between the branch point and 3’ splice site is the 15–20 nucleotide long uridine-rich polypyrimidine tract, which serves as a binding site for U2AF, a core component of the spliceosome. (Will and Luhrmann, 2011). Splicing events rely on complex motif recognition, but the splice sites themselves do not carry sufficient information to define exon boundaries requiring additional regulatory elements for exact exon-intron boundary determination (Lim and Burge, 2001). These regulatory elements include *cis*-elements within the pre-mRNA and associated *trans-*acting splicing factors that trigger or repress splicing with the help of the spliceosome. In addition to *cis-*elements, splicing regulatory proteins recognize other *cis*-regulatory elements. These elements are classified based on their location into exonic and intronic splicing enhancers (ESE and ISE) or exonic and intronic silencers (ESS and ISS) (Wang et al., 2013). While splicing enhancer proteins bind to ESE and ISE sites to reinforce splicing, splicing repressor proteins bind to ESS and ISS sites to repress splicing at nearby sites. Even though intron removal has been described as a deterministic process, Wan *et al.* showed recently that splicing is often a stochastic process lasting from minutes to hours (Wan et al., 2021). Using a combination of high-throughput single-molecule microscopy and deep-sequencing tools, they measured the dynamics of transcription and found that stochastic splicing is more common than previously reported (Sibley et al., 2015).

Interestingly, despite the widespread existence of alternative splicing in human genes, RNAseq studies have demonstrated that many genes express only a single dominant transcript in primary tissues, which are generally referred to as Most Dominant Transcripts (MDT). In an earlier study by Gonzales-Porta *et al.*, 80% of expressed human genes were estimated to have an MDT with an expression at least two-fold higher than the expression of any other minor alternative transcripts (Gonzàlez-Porta et al., 2013). In addition, 50% of expressed genes were found to have the same major transcript across different tissues, while 35% of genes showed switches between major and minor transcripts across different tissues. This finding was indirectly confirmed by Reyes *et al.*, who demonstrated that ∼50% of expressed genes had a tissue-specific transcript (Reyes and Huber, 2018). Moreover, an earlier study found that some genes expressed tissue-specific exons that can play a crucial role in mediating molecular interactions and contributing to signaling pathways (Buljan et al., 2012). The protein product of those tissue-specific transcripts often had disordered regions enriched in protein binding motifs and posttranslational modification sites (Buljan et al., 2012; Ellis et al., 2012) while acting as central protein hubs in protein interaction networks (Bossi and Lehner, 2009).

As different protein isoforms of a gene can have different functions, their combinatorial expression can result in various signaling cascades within cells. Marti-Solano *et al.* demonstrated the combinatorial effect of isoform expression of GPCR (G protein-coupled receptor) proteins in 30 TCGA (The Cancer Genome Atlas) tissues. They found that different gene isoforms had different tissue-expression signatures i.e., combinations of isoform expressions across tissues. As an example, three isoforms of the GPCR gene CNR1 showed four tissue-expression signatures across 30 tissues. In contrast, three isoforms of the GPCR gene CD97 had only one tissue-expression signature in which all isoforms were expressed across all investigated tissues. Thus, the signaling responses downstream of CNR1 seemed to be more tissue-specific and distinct than for CD97 (Marti-Solano et al., 2020).

In another study, Wineberg *et al.* identified 57 differentially expressed cassette exons between epithelial and mesenchymal lineages in kidney development. For example, the *WT1* gene gradually increases the expression of its exon 5 in the development of epithelial cells from mesenchymal lineages resulting in an isoform switching event of WT1 in kidney development (Wineberg et al., 2020).

In summary, alternative splicing is essential for proper cell and tissue differentiation and normal cell function. Its dysregulation is associated with cellular dysfunction causing many diseases like cardiovascular diseases, diabetes, neurological and muscle diseases, immunological and infectious diseases and in particular cancer (Kim et al., 2018; Bonnal et al., 2020; Cherry and Lynch, 2020). In the forthcoming sections, we will describe the mutational and molecular mechanisms underlying dysregulation of alternative splicing with a focus on cancer, give an overview of computational methods to identify and measure these dysregulations and compare the strategies of three recent pan-cancer studies to detect isoform switching events in numerous cancer types. Based on the comparison, we list important points that need to be considered when studying alternative splicing changes. Our recommendations should help to develop new and more robust methodologies for future alternative splicing studies and improve the detection of isoform switching events in cancer.

## Aberrant Alternative Splicing in Cancer

Understanding the relationship between the patterns of alternative splicing and cancer could help to gain insights into the origins of cancer formation and elicit potential therapies targeting cancer-specific protein isoforms (Le et al., 2015; Jaudon et al., 2020; Fuchs et al., 2021; Pan et al., 2021). Aberrant splicing in cancer can be caused by mutations at consensus sequences (5′ splice site, 3’ splice site and branch point), *cis-*regulatory elements (ESE, ESS, ISS, ISE), or mutations and expression changes in genes encoding splicing regulatory proteins. In the following sections, we will describe each of the mutational classes in more detail.

### Mutations in *Cis*-Acting Sequences

Consensus sequences (5′ splice site, 3’ splice site and, branch point) together with *cis-*regulatory elements (ESE, ISS, ESS, ISS) define inclusion or exclusion of exons and introns. Tumor suppressor genes or oncogenes mutated at those sites can have disrupted splicing resulting in gene silencing or activation (DiFeo et al., 2009; Supek et al., 2014; Shiraishi et al., 2018). Interestingly, in a comprehensive large-scale analysis of 31 cancer types from 8,976 samples, ∼50% of *cis*-acting splicing-associated variants were found at non-consensus sequences (Shiraishi et al., 2018). Tumor suppressor genes harbored most of the splicing-associated variants causing exon skipping and alternative splice site usages. Similarly, in another large-scale analysis across 8,656 TCGA tumor samples, recurrent mutations generating alternative splice junctions were identified in various tumor suppressor genes (e.g., *TP53, GATA3, PTEN, SETD2, DDX5, BCOR, SPOP, KDM6A, SMAD4,* and *BAP1*) (Jayasinghe et al., 2018). A recent comprehensive study on TCGA Whole Genome, Exome and RNA Sequencing data showed that 562 mutations in non-coding regions of the human genome created novel splice-site and exon boundaries. Some of these new splice-sites were found in cancer-related genes, such as *TP53, ATRX, BCOR,* and *SMAD4* (Cao et al., 2020), leading to aberrant splicing and functional loss of tumor suppressor genes.

### Alterations in Splicing Factors

Many of the genes that encode core components of the spliceosome and associated regulatory proteins are mutated in cancer (see for a list of genes (Urbanski et al., 2018)). One of the most frequently mutated core components of the spliceosome is the Splicing Factor 3B Subunit 1 (SF3B1). SF3B1 is an essential member of the U2 snRNP core component of the spliceosome complex, anchoring it to the branch point. Recurrent mutations within its C-terminal HEAT (Huntingtin, Elongation factor 3, protein phosphatase 2A, Targets of rapamycin 1) repeat domains have been reported in many cancers, including the blood cancer myelodysplastic syndrome (Malcovati et al., 2011; Papaemmanuil et al., 2011; Yoshida et al., 2011), breast cancer (Fu et al., 2017), and uveal melanoma (Furney et al., 2013; Harbour et al., 2013; Martin et al., 2013). Most cancer-associated mutations in SF3B1 can lead to the usage of an alternative 3′ splice site located upstream of the canonical 3′ splice site (DeBoever et al., 2015) or the usage of an alternative branch point (Darman et al., 2015). Interestingly, alternative 3’ splice sites recognized by mutated SF3B1 are missed in SF3B1 wildtype knockdown or overexpression experiments (Alsafadi et al., 2016). In particular, the SF3B1^K700E^ mutation has been found to reduce intron retention in transcriptomes (Shiozawa et al., 2018), which has also been confirmed by Tang *et al.* using a nanopore sequencing workflow called Full-Length Alternative Isoform analysis of RNA (FLAIR). With their new technology the authors discovered that SF3B1^K700E^ mutations in chronic lymphocytic leukemia globally downregulate intron retentions (Tang et al., 2020).

The other most frequently mutated gene among alternative splicing regulators is Serine/arginine-rich Splicing Factor 2 (*SRSF2*), encodes for a member of the SR-rich *trans-*acting factor protein family. Mutations in *SRSF2* have been reported mainly in hematologic malignancies such as myelodysplastic syndromes, chronic myelomonocytic leukemia, and acute myeloid leukemia (reviewed in (Urbanski et al., 2018)). Almost all mutations in SRSF2 are found at the amino acid position proline 95. Mutations at this site alter the sequence-specific RNA binding activity of SRSF2 resulting in the change of its recognition preference for exonic splicing enhancer recognition motifs (Kim et al., 2015). Thus, mutant SRSF2 can mis-splice the *EZH2* gene, which subsequently undergoes nonsense-medicated decay leading to defects in hematopoietic differentiation (Kim et al., 2015).

### Expression Changes in Splicing Regulators

Up- or down-regulation of splicing regulators are frequently found in acute myeloid leukemia, breast cancer, colorectal adenocarcinoma, and prostate cancer, where 70% of splicing regulators are often upregulated (Sveen et al., 2016). For example, high co-expression levels of 21 splicing factor genes in breast cancer were associated with tumor aggressiveness and high risk for metastasis. One of these splicing factors, hnRNPH, was observed to control the alternative splicing of the RON receptor tyrosine kinase. The upregulation of hnRNPH in gliomas resulted in a switch of the RON protein to a ligand-independent constitutively active isoform (LeFave et al., 2011). As hnRNPH binds sphingosine-1-phosphate lyase 1 (SGPL1), its overexpression causes the stabilization and upregulation of SGPL1 in colorectal cancer cells, thereby inhibiting apoptosis and promoting tumor progression (Takahashi et al., 2020). Interestingly, splicing factors in breast cancers tend to be hit by copy number alterations and associated expression changes rather than recurrent mutations (Park et al., 2019).

The majority of chromophobe renal cell carcinomas, on the other hand, show a somatic copy number loss and associated loss of *SF3B1* expression (Paolella et al., 2017). However, in related cancer types of clear cell and papillary renal cell carcinomas (Paolella et al., 2017; Ohashi et al., 2019), SF3B1 tends to be overexpressed as well as in other cancer types such as hepatocellular carcinoma leading to lower survival rates (López-Cánovas et al., 2021).

PRPF6, a U5 snRNP, is another splicing factor frequently overexpressed in cancer cell lines, including colorectal carcinoma leading to the aberrant splicing of the oncogenic form of the mitogen-activated protein kinase 20 (MAP3K20) (Adler et al., 2014).

## Computational Tools and Resources to Detect Differential Transcript Usage and Its Functional Impact

To detect the aforementioned splicing events, many computational software packages and websites have been developed for differential splicing analysis. They can be classified into count-based methods and isoform-based methods, the former which can be further divided into exon-based methods and event-based methods. Software tools among the exon-based methods are for example DESeq2 (Love et al., 2014), DEXSeq (Anders et al., 2012), edgeR (Robinson et al., 2010; Anders et al., 2012), JunctionSeq (Hartley and Mullikin, 2016), limma (Ritchie et al., 2015), while event-based methods include dSpliceType (Zhu et al., 2015), MAJIQ (https://majiq.biociphers.org), rMATS (Shen et al., 2014), SUPPA2 (Trincado et al., 2018), and LeafCutter (Li et al., 2018). Example for isoform-based methods include Cuffdiff2 (Trapnell et al., 2013) and DiffSplice (Hu et al., 2013). Exon-based methods compare read counts at exons or exon-junctions between different conditions, while event-based methods compare the percentage of spliced-in values of splicing events such as intron retention and exon skipping between conditions (Muller et al., 2021). On the other hand, isoform-based methods align the collection of all paired-end reads to the full-length sequence of each isoform to model their abundances. In a recent comparative study, Mehmood A. *et al.* evaluated the performance of 10 differential splicing analysis tools, including exon-based, event-based, and isoform-based methods on four different vertebrate data sets. They measured the tools’ consistency, reproducibility, precision, recall, and false discovery rate and found that all exon-based methods outperformed the other tools in identifying qPCR-validated differential splicing events. However, overall, the performances varied according to different data sets, why the authors emphasized running multiple tools in differential splicing analysis projects (Mehmood et al., 2020).

**FIGURE 2:**
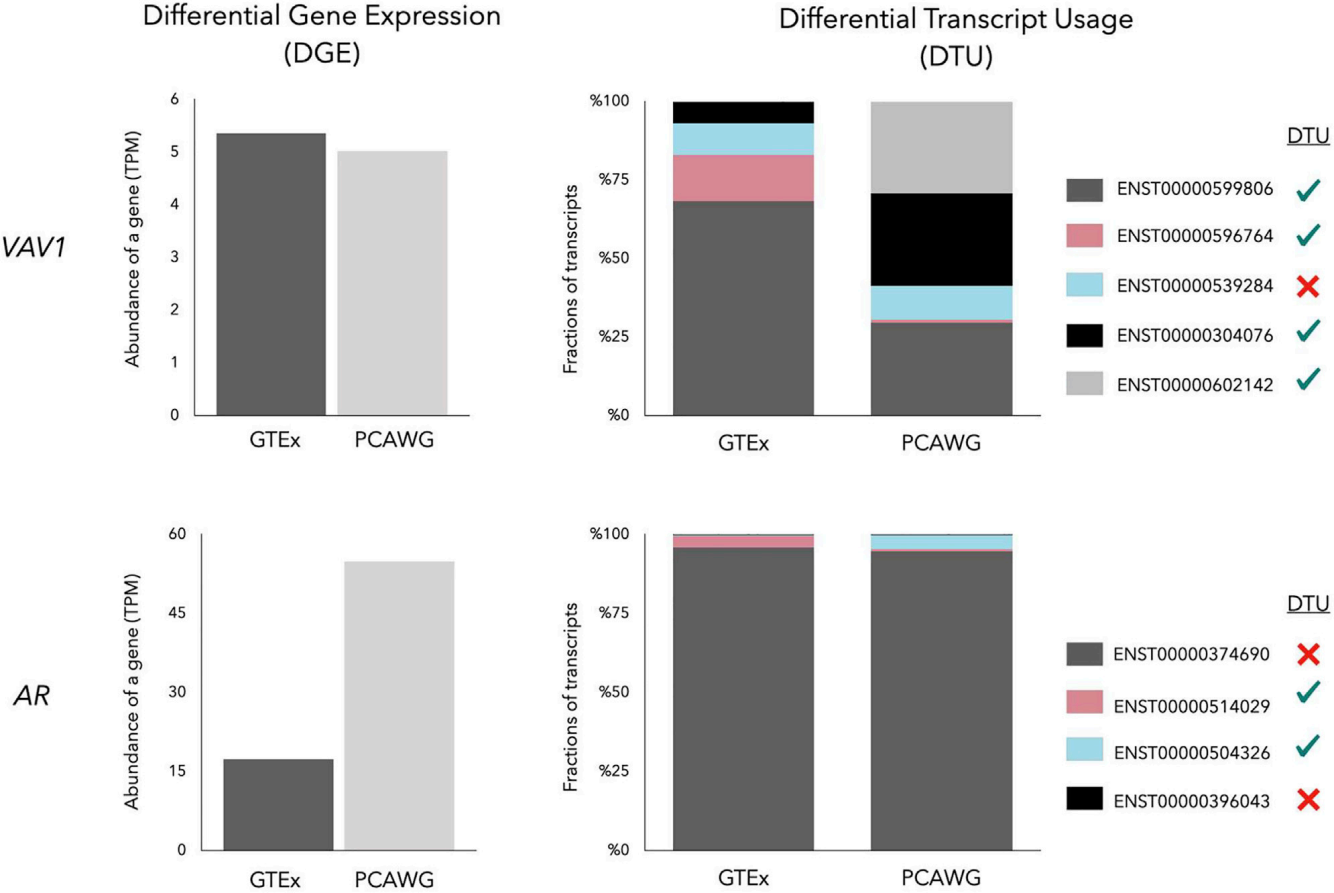
Gene Expression of *VAV1* and *AR* in a prostate normal tissue (GTEx) and a prostate cancer (PCAWG) sample. While the *VAV1* gene has a similar total expression, the *AR* gene is overexpressed in prostate cancer showing Differential Gene Expression (DGE). Despite the similar total gene expression, four of five transcripts of *VAV1* undergo Differential Transcript Usage (DTU) between normal and prostate cancer, symbolized by four green ticks and one red cross in the legend. The major transcript of AR (dark grey bar) remains highly expressed in prostate cancer, but minor transcripts, including AR-V7 (blue bar), are subjected to DTU.

In contrast to differential expression analysis, methods that measure Differential Transcript Usage (DTU) test the significance of relative abundance changes of transcripts in different conditions. DTU can provide complementary information to Differential Gene Expression (DGE) analysis (Figure 2). Genes with the same total expression level in different experimental conditions might have a different predominantly expressed transcript. There are various Bioconductor packages available for DTU analysis such as DEXSeq (Anders et al., 2012), diffSpliceDGE (Robinson et al., 2010), diffSplice (Hu et al., 2013), DRIMSeq (Nowicka and Robinson, 2016), BANDITS (Tiberi and Robinson, 2020), IsoformSwitchAnalyzeR (Vitting-Seerup and Sandelin, 2019) and TSIS (Guo et al., 2017). These methods have been developed for bulk RNA-seq data. Their scalability with single-cell RNA-seq (scRNA-seq) data is limited. Recently, Gilis *et al.* assessed different DTU methods on both bulk and scRNA-seq simulated data and concluded that many DTU methods are unable to handle large volumes of data (e.g., 30,000 transcripts), prolonging the analysis to several days (Gilis et al., 2021). To compensate for this bottleneck the authors developed SatuRn, which uses flexible quasi-binomial generalized linear modeling to enhance DTU analysis (Gilis et al., 2021). The computational pipeline Sierra (Patrick et al., 2020), on the other hand, applies a splice-aware peak calling algorithm based on DEXSeq to cope with massive polyA-captured scRNA-seq data.

Besides detecting DTU, IsoformSwitchAnalyzeR identifies functional consequences including intron retention, open reading frame, nonsense-mediated decay sensitivity, coding potential etc. (Vitting-Seerup and Sandelin, 2019). It is dependent on other tools for transcript abundance calculations. Similarly, TappAS is a new computational framework to calculate DTU and study differential polyA site usage and analysis of UTR lengths between isoforms (de la Fuente et al., 2020).

ISOexpresso is a web server on isoform switching events that performs live comparisons between isoform expression levels of different TCGA cancer types and matched normal samples (Yang et al., 2016). The domain changer presenter (DoChaP) is a web server that visualizes exon and protein-domain differences between all transcripts of a gene (Gal-Oz et al., 2021). The Domain Interaction Graph Guided ExploreR (DIGGER) database, on the other hand, provides protein-protein and domain-domain interaction visualization for known protein isoforms, giving hints to which interactions are lost or preserved in different isoforms (Louadi et al., 2021). We have recently developed CanIsoNet, a web server, to study the pathogenic impact of cancer-specific most dominant transcript in multiple cancer types (Karakulak et al., 2021). All the computational tools and resources mentioned above serve as a gateway to better understand the functional impact of alternative splicing events.

In recent years, additional strategies have been developed to study isoform switching events in pan-cancer studies. In the next section, we will discuss these strategies in detail and highlight the similarities and differences in their results.

## Comparison of Pan-Cancer Studies to Detect Isoform Switching Events

Pan-cancer studies aim to dissect genomic and transcriptomics similarities and differences between various cancer types. Over the last 4 years, three large pan-cancer projects have utilized large-scale sequencing data from The Cancer Genome Atlas (TCGA) (The Cancer Genome Atlas Research Network et al., 2013), and the Pan-Cancer Analysis of Whole Genomes (PCAWG) projects (The ICGC/TCGA Pan-Cancer Analysis of Whole Genomes Consortium, 2020) to analyze isoform switching events and their functional impact in multiple cancer types. The first two studies covered 4,542 and 5,500 different cancer samples from 11 to 12 different cancer types, respectively. The former was performed by Climente-González *et al.* (Climente-González et al., 2017) while the latter was conducted by Vitting-Seerup and Sandelin (Vitting-Seerup and Sandelin, 2017). Recently, our lab published the most comprehensive study in terms of cancer types covering 27 cancer types, including various subtypes with a total of 1,209 cancer samples (Kahraman et al., 2020).

Interestingly, all three projects report different number of isoform switching events (see Table 1). The Vitting-Seerup study discovered 4,446 cancer-specific isoform switching events (2,792 unique isoform switching events) from 2,352 genes across 12 cancer types (Vitting-Seerup and Sandelin, 2017). Each splicing event was predicted to have a functional impact due to changes in protein domain structures. The Climente-González study detected 37,476 cancer-specific isoform switching events (8,122 unique isoform switching events) from 6,442 genes (Climente-González et al., 2017) across 11 different cancer types while the Kahraman study discovered over 31,748 cancer-specific isoform switching events (13,498 unique isoform switching events) from 7,143 genes across 27 different cancer types (Kahraman et al., 2020).

**TABLE 1:** Technical details of recent pan-cancer studies on isoform switching events. Abbreviations: CI = Confidence Interval, FDR = False Discovery Rate, FPKM = Fragments Per Kilobase of transcript per Million mapped fragments, GRCh37 = Genome Reference Consortium human build version 37, IF = Isoform Fraction, MDT = Most Dominant Transcript, PCAWG = Pan-Cancer Analysis of Whole Genomes, PSI = Percent-Spliced-In, RLE = Relative Log Expression, TCGA = The Cancer Genome Atlas, TPM = Transcripts Per Million. Definitions of cancer types’ abbreviations can be found in the abbreviation list at the end of the manuscript.

	Climente-Gonzales *et al.*	Vitting-Seerup *et al.*	Kahraman *et al.*
Data source	TCGA	TCGA	PCAWG
Data Set	4,542 cancer samples from 11 solid cancer types	5,562 cancer samples from 12 solid cancer types	2,232 GTEx normal samples and 1209 cancer samples from 27 solid and hematological cancer types.
Cancer Types	BRCA, COAD, KICH, KIRP, KIRC, LIHC, LUAD, LUSC, PRAD, THCA, HNSC	BRCA, COAD, KICH, KIRP, KIRC, LIHC, LUAD, LUSC, PRAD, THCA, STAD, HNSC	BRCA, COAD, KICH, LIHC, LUAD, LUSC, PRAD, STAD, THCA, HNSC.
			In addition: Kidney-RCC(KIRP, KIRC), Biliary-AdenoCA, Bladder-TCC, Bone-Leiomyo, Breast-LobularCA, Cervix-AdenoCA, Cervix-SCC, CNS-GBM, CNS-Oligo, Eso-AdenoCA, Lymph-BNHL, Lymph-CLL, Lymph-NOS, Ovary-AdenoCA, Panc-AdenoCA, Skin-Melanoma, Uterus-AdenoCA
Genome Assembly	GRCh37	GRCh37	GRCh37
Library Filtering	—	• Discard samples with <20 million reads	• Only Samples labeled as whitelisted in the PCAWG project
		• One library per patient	• Discard cancer types with ≤2 samples
		• Only cancer types with ≥25 paired samples	—
RNA-seq data processing	• TPM for PSI calculation	• RLE normalized FPKM values for IF calculation	• TPM values for MDT calculations
	• Transcripts with TPM <0.1 were ignored		• Transcripts with TPM<2 in PCAWG and TPM<0.2 in GTEx were ignored
Isoform Switch Calculation	• Genes containing multiple isoforms	• Genes containing multiple isoforms	• Genes containing multiple isoforms
	• Discard genes outside of 95% CI of normal expression values	• Discard isoforms and genes if 95% CI of mean expression <1 FPKM	• Compute MDT: 1st ranked transcript expression ≥ 2 × 2nd ranked transcript expression
	• Discard differentially expressed genes (Wilcox-Rank Sum test *p*-value < 0.01)	• dIF = IF_tumor_—IF_normal_	• Discard transcripts found as MDT in GTEx
	• dPSI = PSI_tumor_ –PSI_normal_	• For paired samples in a cancer type, compute mean of all paired sample dIF	• Discard genes not having an MDT in 50% of GTEx samples
	• Ignore cancer cases with PSI_tumor_ < PSI_normal_ and normal cases with PSI_normal_ < PSI_tumor_	• Also compute between all cancer and normal samples difference in mean dIF	• Discard transcripts in a cancer sample, if relative expression is smaller than median expression in GTEx.
	• Discard lowly recurrent switches (binomial test, adjusted *p* < 0.05)	• Significant isoform switch if mean dIF>0.1 and FDR<0.05.	• Cancer-specific MDT if FDR corrected sign-test<0.01.
	• Significant isoform switch if dPSI>0.05 and empirical *p* < 0.01.	—	—
Statistical Tests	Empirical *p*-value computed by comparing dPSI of a cancer sample to distribution of dPSI values from all pairwise normal samples.	Benjamini–Hochberg	Benjamini–Hochberg
		FDR corrected paired-Mann-Whitney U test for paired samples, Unpaired-Mann-Whitney U test for unpaired samples	FDR corrected sign test, in which a transcript’s relative expression in a cancer sample is compared to all GTEx expression values and the frequency of higher and lower values is tested using two-sided bionomial test.
Unique Isoform Switching Events	8,122 from 6,442 genes	2,792 from 2352 genes	11,040 from 7,143 genes
Total Isoform Switching Events Across Different Cancer Types	37,476	4,446	31,748

With respect to the methodology, each of the three studies used slightly different definitions for an isoform switching event: the Climente-González study computed *differential transcript isoform usage* by comparing Transcript Per Million (TPM) values of transcripts in tumor and normal samples from TCGA. A Proportion Spliced-In (PSI) score assessed the relative expression of a transcript with respect to the total gene expression. A differential PSI score defined as dPSI = PSI_tumor_–PSI_normal_ estimated the differential transcript isoform usage. Where possible, the matched normal samples were used, or if absent, the median PSI of a transcript in all normal samples across the same tissue was utilized. Genes displaying differential expression were discarded to avoid misleading results in the study.

The Vitting-Seerup study took a similar approach using the same TCGA data. However, they used Relative Log Expression (RLE) normalized Fragments Per Kilobase Million (FPKM) counts and considered only isoform switching events that had been detected in both matched and unmatched tumor samples (Table 1). In contrast to Climente-González *et al.*, relative expression values of the transcripts were termed Isoform Fraction (IF) and used to compute a dIF score which compares the difference in the IF values of transcripts between cancer and normal samples. A minimum number of 25 isoform fraction values per condition was required for calling an isoform switch event.

The Kahraman study, identified isoform switching events by comparing Most Dominant Transcripts (MDTs) within the PCAWG project. Note, that over 50% of PCAWG’s RNA-seq samples were originating from TCGA. An MDT was defined as a transcript whose expression value was at least two-times higher than the second most expressed alternative transcript of the same gene in the same sample. As expression values from matched normal samples were mostly not available in PCAWG, the Kahraman study used expression values from the Genotype-Tissue Expression (GTEx) project (Lonsdale et al., 2013). The GTEx project stores gene and transcript expression information for 54 tissue types collected from nearly 1,000 individuals. A cancer-specific MDT (cMDT) was called, if an MDT in a cancer sample was unique to the cancer and not observed as an MDT in the matched GTEx tissue type. Similar to the Climente-González study, Kahraman *et al.* calculated isoform switching events per-patient.

To reduce the number of false-positive identification, the Climente-González study computed an empirical *p*-value for each isoform switch using its dPSI value in comparison to the distribution of dPSI values in normal samples. Each isoform was required to have a dPSI >0.05 and a *p*-value <0.01. In contrast, the Vitting-Seerup study compared the dIF values of matched samples for each cancer type using a paired Mann–Whitney U test. To generalize their results, all dIF values in a cancer type were also compared with a standard Mann–Whitney U test to all dIF values in normal samples. Genes having at least one transcript with dIF >10% and FDR corrected *p*-value < 0.05 were regarded as having an isoform switching event. The Kahraman study used various filters and a sign-test to detect significant isoform switching events. The filters included the uniqueness of a cMDT to a cancer type, the requirement of a cMDT gene to have an MDT in ≥50% of the matched GTEx samples, and a higher relative expression of a cMDT than the median relative expression in the matched GTEx cohort. To test for significant hits, a sign-test was utilized comparing the relative expression of cMDT to its expression values in the matched GTEx cohort. cMDTs were required to have Benjamini–Hochberg FDR corrected *p*-value < 0.01.

**FIGURE 3:**
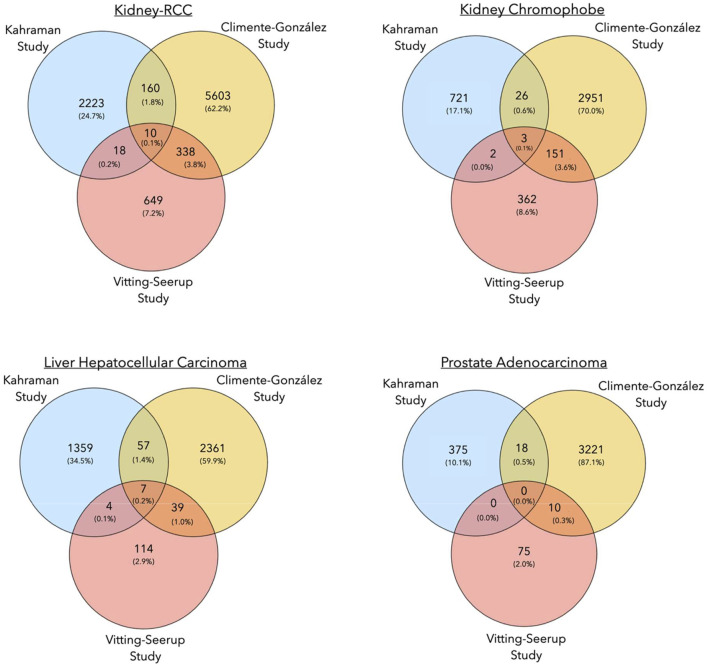
Venn diagrams showing the number of common isoform switching events in three pan-cancer studies for Kidney-RCC, kidney chromophobe, liver hepatocellular carcinoma and, prostate adenocarcinoma.

To understand to what extent the results of the three pan-cancer studies resemble each other, we compared the cancer-specific transcripts reported by each study. Overall, the overlap between genes undergoing isoform switching events and cancer-specific isoforms between all three studies was small (Supplementary File S1, Table 2, Table 3). The highest number of common events were detected for Kidney-RCC and Liver hepatocellular carcinoma with 10 and 7 common switches, respectively (Super Exact Test *p*-value: < 4.2 × 10^−6^), while no overlap was found for prostate adenocarcinoma and thyroid carcinoma (Figure 3; Table 2).

**TABLE 2:** Common isoform switching events between the studies of Climente-González *et al.*, Kahraman *et al.* and Vitting-Seerup *et al.*

Cancer Types	Isoform Switching Events
	Normal Isoform_Cancer-specific Isoform (Gene Name)
BRCA	uc003gbj_uc003gbi (CPLX1)
	uc004aso_uc004asp (BICD2)
	uc003ncb_uc011djb (CAP2)
COAD	uc002vhm_uc002vhq (PNKD)
KICH	uc002otv_uc002otw (CEACAM1)
	uc001mvx_uc001mvw (CD44)
	uc002unj_uc002unn (ZNF385B)
LIHC	uc003iew_uc003iex (NUDT6)
	uc003tbv_uc010kwf (AQP1)
	uc003pko_uc010kbr (NT5E)
	uc003tre_uc003trf (NIPSNAP2)
	uc002yki_uc002ykj (CXADR)
	uc001kgn_uc001kgo (PANK1)
	uc001qyu_uc001qyt (YBX3)
LUAD	uc003gbj_uc003gbi (CPLX1)
	uc002yki_uc002ykj (CXADR)
LUSC	uc002uxj_uc010ftb (CASP10)
	uc002nym_uc002nyn (LSR)
	uc003vqg_uc003vqc (MEST)
PRAD	No common isoform switching event
THCA	No common isoform switching event
KIRC and KIRP	uc001kza_uc001kyy (MXI1)
	uc003akb_uc003ake (RNF185)
	uc004evg_uc004evi (AIFM1)
	uc002bdb_uc002bdc (CIB2)
	uc002cok_uc002coi (SLC9A3R2)
	uc001dpx_uc001dpy (BCAR3)
	uc002yki_uc002ykj (CXADR)
	uc004bsg_uc004bsi (FBGS)
	uc003aks_uc003akr (PATZ1)
	uc003eas_uc003eaq (GRAMD1C)

**TABLE 3:** List of common genes having an isoform switching event in Climente-González *et al.*, Kahraman *et al.*, Vitting-Seerup *et al.*

Overlaps of 3 Studies	Gene Names
BRCA	*TNC, ATXN3, FAM76B, CAP2, PNPLA7, PPAN, DACH1, CPLX1, SKA2, BICD2, HOXC6*
COAD	*C19orf60, OSBPL5, CD44, ZFYVE16, TLE2, ST6GALNAC1, LIMS2, CALD1, PNKD, SHC2, RIN2, MYO10, C7orf50, AK3, RABGEF1, TSPAN7, FMNL3, ING2, PCCA, SH3BGR, BTN3A2*
KICH	*CD44, RC3H2, CEACAM1, NRCAM, CDADC1, GSPT1, CAV1, DNMBP, LIPA, NCOA7, KTN1, PDGFRA, BCAR3, RGS3, TARS2, ZNF385B, OBSCN, NAGS, BSCL2, PCMTD1, AP1S2, EXD3, ZNF44, WWP2, CTNND1, SCAMP5*
LIHC	*KIF22, NXT2, NT5E, GBAS, PANK1, CXADR, MFAP4, NUDT6, FGGY, AQP1*
LUAD	*SPAG9, LSR, ITM2C, CXADR, CPLX1, TNFRSF10C*
LUSC	*CASP10, SDCCAG8, ATXN3, CECR1, DYRK1B, LSR, MEST, GAB1, CNOT2, MKLN1, SHROOM2, NTRK2, DST, IL17RE, PPFIBP2, WWOX, CTNND1*
PRAD	*VAMP1*
THCA	*CHF, Clorf198, FAM174B*
KIRC + KIRP	*C19orf60, MLXIPL, PLEKHH1, ATP2B4, SLC9A3R2, FGFR2, RASSF1, ATP2B1, BAZ2A, SMARCA2, EPS15, IPO11, MMP2, CECR1, MFNG, PATZ1, ASB9, FLT1, TAF1C, ILVBL, TIMM50, ADAP1, CAV1, DOCK8, DNMBP, LIPA, SHOC2, KLHL2, ST3GAL4, MVK, SCNN1A, BTN3A3, DOCK7, MXI1, ARAP3, ACOT9, IQSEC2, PSD4, EML2, TRPM4, EPS8L1, DIAPH1, DMGDH, PDGFRA, BIVM, ITM2C, CIB2, BIN1, FPGS, BCAR3, NCOA4, RNF185, SSBP2, DCDC2, ZNF185, TM7SF2, SCOC, ABI3BP, CXADR, RABGEF1, RMND1, AIFM1, SLC35B2, KALRN, MUM1, TBC1D24, STK36, HDAC11, MYO5B, TMCC1, GOLGA8A, EFCAB4A, GRAMD1C, LIMK2, SPNS3, AFMID, RBM33, FAM174B, MITF, ZNF559, TMEM201, ZNF44, WWP2, SCAMP5, PPME1, CUX1*

Gene names are shown in italics.

The overlap of isoform switching events between the Climente-González and Vitting-Seerup studies was higher (Supplementary File S2). For the prostate cancer cases mentioned above, we identified a total of ten common isoform switches between both studies (Super Exact Test, *p*-value: 4.6 × 10^−4^). The largest overlaps with 154 and 348 common transcript switches between both studies were found for kidney chromophobe cancer and again for Kidney-RCC, respectively (Super Exact Test *p*-value: < 4.2 × 10^−6^) (Figure 3; Table 2). In general, the higher number of isoform switching events in the Climente-González study (37,476 in total) compared to the Vitting-Seerup study (4,446 in total) seemed to be related to the less stringent criteria for detecting switching events; Climente-González dPSI ≥0.05 vs Vitting-Seerup dIF ≥0.1 (Table 1).

The paucity of common transcripts detected as switch events in both studies and the Kahraman study is most likely due to lack of matched normal samples in the latter study. Contributing to the difference might also be contaminations in GTEx that were found in highly expressed and tissue-enriched genes (Nieuwenhuis et al., 2020). The Kahraman study addressed these issues by applying various rigorous filters and statistical tests and by focusing only on MDT calls. In addition, the Kahraman study used Ensembl (Hubbard, 2002) as a transcript annotation database, while the other two studies used the UCSC database. As a result, only 11,495/13,498 of unique isoform switching events were matched between the databases. Lastly, the smaller number of isoform switches reported by the Vitting-Seerup study (4,446 in total) could be traced back to the focus of the study on isoform switches with functional consequence only.

## Discussion

There were two main drawbacks in our comparison of the three pan-cancer studies. Firstly, to the best of our knowledge, a gold-standard data set with experimentally verified isoform switching events in various cancer types was not available for our comparison. Without a controlled setting in which only the parameters of the applied methods are varied, the identification of the strengths and weaknesses of methods is difficult to recognize. Secondly, the methods behind the pan-cancer studies were not readily available as software packages. Instead, all three methods were loose collections of different software tools connected with custom scripts into analysis pipelines, tailored for their particular data sets. Thus, it was not possible to run the three methodologies on an identical data set for a thorough assessment of their performances.

Despite the difficulties, our comparison of the three pan-cancer studies revealed only a small number of common switching events between the different methods. Given the discrepancy in the results of the three pan-cancer studies following important points should be considered for any isoform switching analysis:

• Matched *vs* unmatched normal samples: Whenever possible, matched normal samples should be used. If matched normal samples are not available, GTEx data are an alternative. In the latter case, potential biological artefacts should be controlled as covariates such as gender, age, tissue type etc. (Wang et al., 2018). Notably, the same quantification analysis pipeline should be used for cancer and GTEx samples (Zeng et al., 2019).
• Redundant cDNA sequences: Some protein isoforms have identical cDNA sequences with different translation start sites, e.g., TP53-206 and TP53-220 (Ensembl Database v104). The detection of a single most dominant transcript for such genes is not trivial. Depending on the analysis pipeline, the user might want to remove redundant cDNA sequences to improve the detection of most dominant transcripts.
• Most Dominant Transcripts vs Isoform Fraction: Isoform switching events can be determined by detecting and comparing Most Dominant Transcripts (MDT) or Isoform Fractions between different conditions. As many genes have a single most dominant transcript under normal conditions (Ezkurdia et al., 2015), the identification of MDT switches is a reasonable approach for detecting cancer-specific alternative splicing events. However, significant changes in the expression of minor transcripts can be missed in an MDT analysis (see next point). Under such circumstances, the usage of isoform fractions can be more appropriate for splicing analysis. A critical step in isoform fraction analysis is deciding a proper cut-off for detecting isoform switching events; the lower the cut-off, the higher the risk for false-positive identifications.
• Minor transcripts: Changes in the expression of minor transcripts can be as important as most dominant transcript switches. For example, the V7 transcript of the Androgen Receptor (AR-V7) is a constitutively active nuclear receptor found primarily overexpressed in metastatic castration-resistant prostate cancers (mCRPC) (Tan et al., 2015; Zhang et al., 2020). The overexpression of AR-V7 emerges as a resistance mechanism to androgen deprivation therapies and is used to switch the treatment of prostate cancer patients from an AR inhibitor to a standard of care chemotherapy (Graf et al., 2020). Despite the over-expression of AR-V7, the main expressed AR transcript remains the canonical full-length AR transcript. Therefore, focusing on the most dominant transcript of AR only would miss significant expression changes in AR-V7.
• Transcript Count Normalization Methods: Normalization of raw RNA-seq expression data is crucial for addressing biases within-samples (e.g., length of a gene, GC content), and between-samples (e.g., sequencing coverage, total RNA yield, batch effects) (Evans et al., 2018). FPKM (Fragments Per Kilobase of transcript per Million fragments mapped) and TPM (transcripts per million) are often used as normalizations methods. RPKM and FPKM have been primarily developed to account for within-sample biases. On the other hand, TPM takes average invariances into account. As the sum of all TPM values in different samples is the same, the TPM measure should be used whenever the expression values of different samples are compared, e.g., PCAWG *vs* GTEx samples.
• Statistical Methods: A paired or unpaired Mann-Whitney U test is ideal for testing significant alternative splicing changes between matched or unmatched cohorts, respectively, like in the Vitting-Seerup study. On the other hand, if patient-specific differences between isoform expressions should be detected, variations of a binomial test applied by Climente-González *et al.* and Kahraman *et al.* should be used. Climente-González used the binomial test to filter out isoforms switches that are not recurrent across different cancer samples. The Kahraman study applied a sign-test with a two-sided binomial test to compare the expression value of a single cancer sample to the matched GTEx cohort.

A general problem of the three projects mentioned above is their usage of short-read sequencing data to identify full-length isoform sequences. Wang *et al.* identified large differences in the RNA and protein isoform sequences compared to RefSeq and Ensembl in rat hippocampus by using full-length RNA sequencing technology in combination with polysome profiling and ribosome footprinting (Wang et al., 2019). Thus, the application of long-read third-generation sequencing (TGS) technologies (Pacific BioScience and Oxford Nanopore) should be prioritized for future isoform-specific alternative splicing studies. Furthermore, 40% of alternatively spliced transcripts include premature termination codons, which are degraded by the non-sense mediated decay pathway (Tabrez et al., 2017). The degradation is part of the reason why the correlation between transcript expression and protein expression is often below *R* ^2^ < 0.4 in multi-cellular organisms (de Sousa Abreu et al., 2009; Schwanhäusser et al., 2011). Under such circumstances, changes in transcript expression detected in isoform switch analysis can be buffered out on the proteome level. Thus, it is important to validate potential transcript biomarkers using proteomics approaches to ensure that the effect of distinct transcript expressions unfolds on the proteome and cellular level. This is especially true for identifying putative splicing-derived neoantigens for immunotherapy decisions (Kahles et al., 2018).

With new developments of technologies to detect full-length transcripts and proteins isoforms and the implementation of new computational methods and databases to analyze these data sets, we hope that future studies will further highlight the importance of alternative splicing to disease development and lead to new targeted therapies against disease-causing splicing events.

## Methods

To understand to what extent the results of the pan-cancer studies of Climente-González *et al.*, Vitting-Seerup *et al.*, and Kahraman *et al.* resemble each other, we collected four different statistics on the reported isoform switch events per cancer type.

1) Number of common genes having an isoform switching event
2) Number of common transcripts from normal samples found in isoform switching events
3) Number of common transcripts from cancer samples found in isoform switching events
4) Number of overlapping isoform switching events where transcripts from normal samples are identical and transcripts from cancer samples are identical.

The statistics were assessed only for the cancer types BRCA, COAD, KICH, LIHC, LUAD, LUSC, PRAD, THCA, Kidney-RCC (KIRC + KIRP), which were shared between all three studies.

The Climente-González and the Vitting-Seerup studies used the UCSC identifiers for isoform annotations, while the Kahraman study used Ensembl Transcript IDs (ENST IDs). Thus, we first matched 247,540 UCSC Transcript IDs to ENST IDs via a mapping table from the UCSC database. We compared the gene and isoforms identifiers of all cancer-specific isoforms from the three studies using the ggvenn package of ggplot2 R library (Wickham, 2016) and the SuperExactTest package (M. Wang, 2015) (please see Github repository). The ggvenn library was used to draw Venn diagrams for each overlap calculation. The SuperExactTest package was used to compute the significance of overlaps using the Super Exact significance test, which is related to Fisher’s Exact test but applicable to multiset intersections and data sets. R and Python scripts for calculating data set overlaps can be found at https://github.com/KarakulakTulay/Isoform_Comparison.

## Glossary

AdenoCA: adenocarcinoma
Bladder-TCC: bladder transitional cell carcinoma
Bone-Leiomyo: bone/soft Tissue leiomyosarcoma
BRCA: breast invasive carcinoma
Cervix-SCC: cervix squamous cell carcinoma
cMDT: cancer-specific most dominant transcript
CNS-GBM: CNS glioblastoma
CNS-Oligo: CNS oligodendroglioma
COAD: colon adenocarcinoma
ColoRect-AdenoCA: colon/Rectum adenocarcinoma
DGE: differentially expressed genes
DTU: differential transcript usage
ESE: exonic splicer enhancer
Eso-AdenoCA: esophagus adenocarcinoma
ESS: exonic splicer silencer
GTEx: genotype-tissue expression
Head-SCC: head/neck squamous cell carcinoma
HNSC: head and neck squamous cell carcinoma
ISE: intronic splicer enhancer
ISS: intronic splicer enhancer
KICH: kidney chromophobe
KIRC: kidney renal clear cell carcinoma
KIRP: kidney renal papillary cell carcinoma
Kidney-RCC: kidney renal cell carcinoma, clear cell and papillary
LIHC: liver hepatocellular carcinoma
LobularCA: lobular carcinoma
LUAD: lung adenocarcinoma
LUSC: lung squamous cell carcinoma
Lymph-BNHLl: ymphoid mature B-cell lymphoma
Lymph-CLL: lymphoid chronic lymphocytic leukemia
Lymph-NOS: lymphoid–not otherwise specified
Panc-AdenoCA: pancreas adenocarcinoma
PCAWG: pan-cancer analysis of whole genomes
PRAD: prostate adenocarcinoma
STAD: stomach adenocarcinoma
TCGA: the cancer genome atlas
THCA: thyroid carcinoma
