# Supplementary material for: Probing Isoform Switching Events in Various Cancer Types: Lessons From Pan-Cancer Studies
Source: Front Mol Biosci. 2021 Nov 23;8:726902. doi: 10.3389/fmolb.2021.726902 (PMC8650491; doi:10.3389/fmolb.2021.726902)

Supplementary information

Probing Isoform Switching Events inVarious Cancer Types: Lessons From Pan-Cancer Studies

Tülay Karakulak, Holger Moch, Christian von Mering and Abdullah Kahraman

SuperExactTest* analysis of three PanCancer Studies

**See Methods section in the manuscript.*
In the following figures, numbers next to the circles show the sizes of each dataset. The green circles show presence of the dataset in the overlap analysis. The intensity of the colour represents the significance (p-value) of overlap between datasets.

Isoform Switch Analysis

Breast Invasive Carcinoma (BRCA)

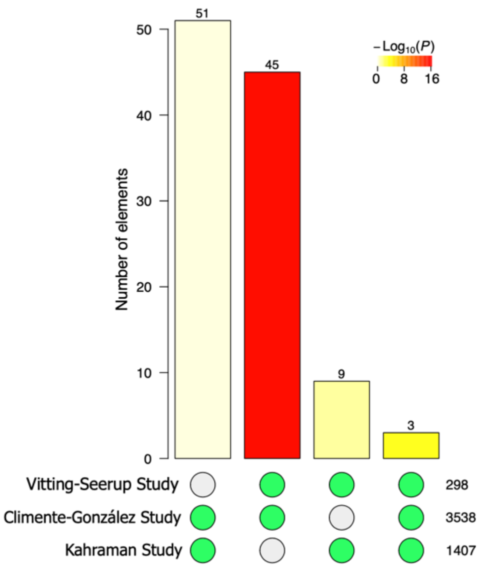

Colon Adenocarcinoma (COAD)

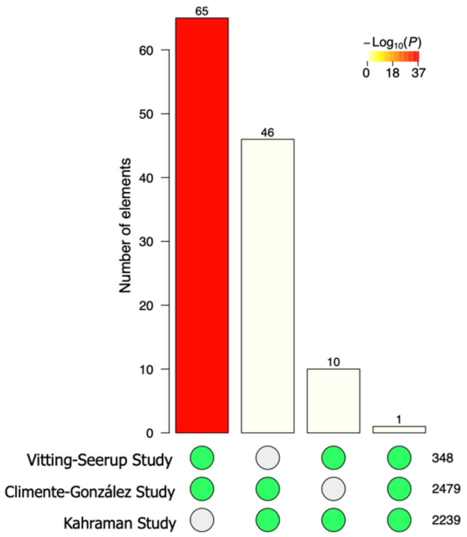

Kidney Chromophobe (KICH)

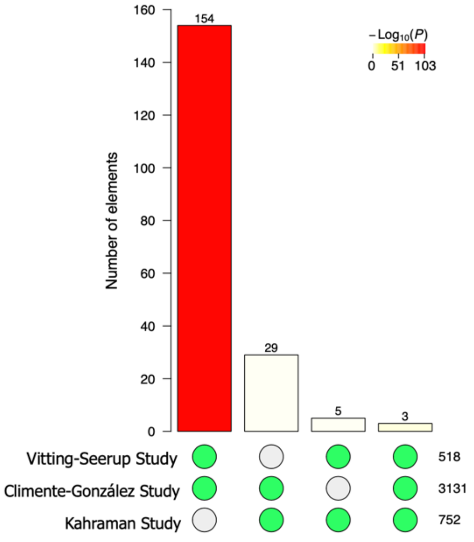

Liver Hepatocellular Carcinoma (LIHC)

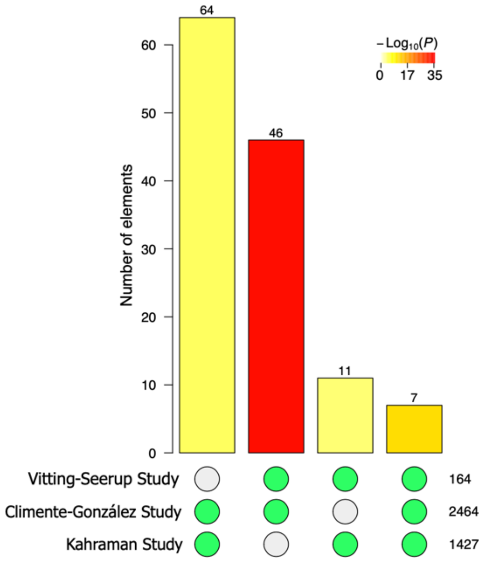

Lung Adenocarcinoma (LUAD)

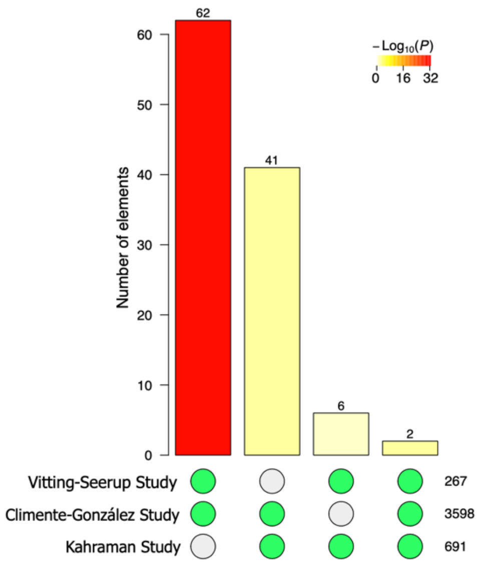

Lung Squamous Cell Carcinoma (LUSC)

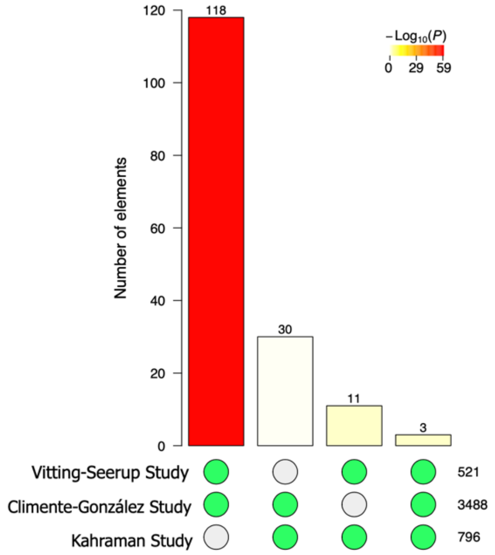

Prostate Adenocarcinoma (PRAD)

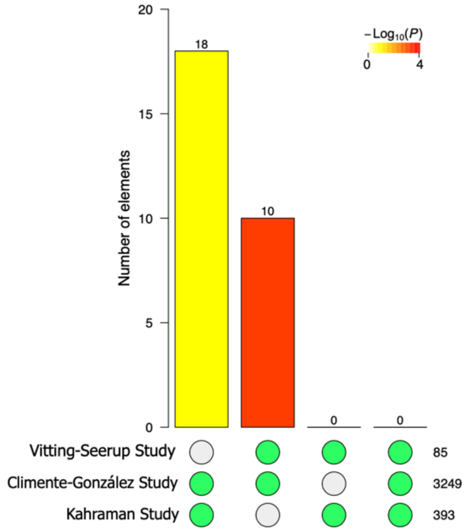

Thyroid Carcinoma (THCA)

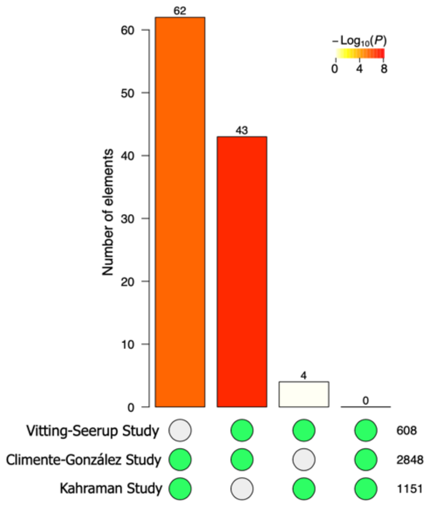

Kidney Renal Cell Carcinoma, Clear Cell and Papillary (KIRC + KIRP)

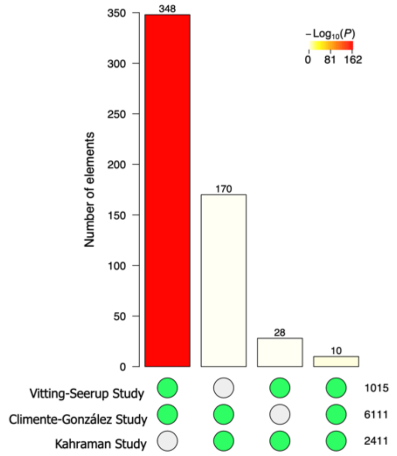

Gene Set Analysis

Breast Invasive Carcinoma (BRCA)

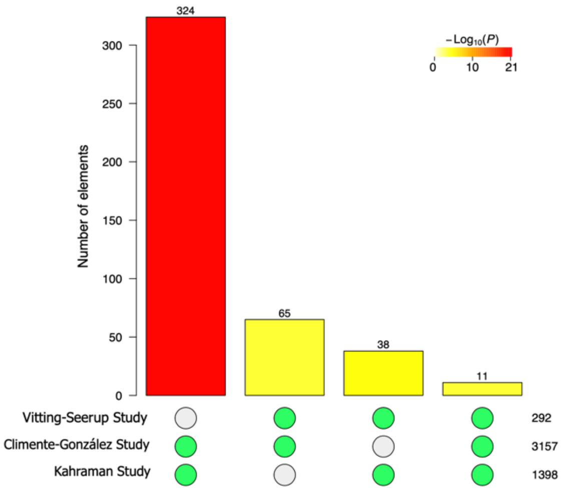

Colon Adenocarcinoma (COAD)

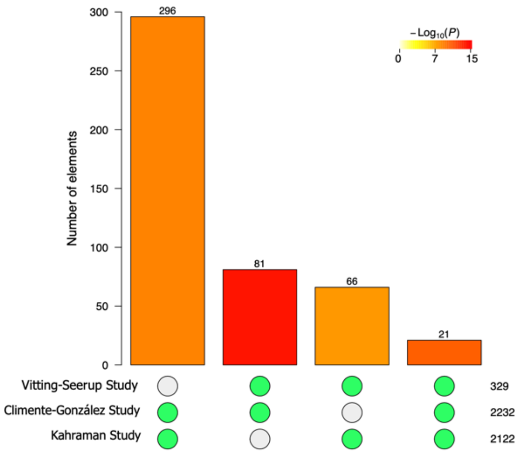

Kidney Chromophobe (KICH)

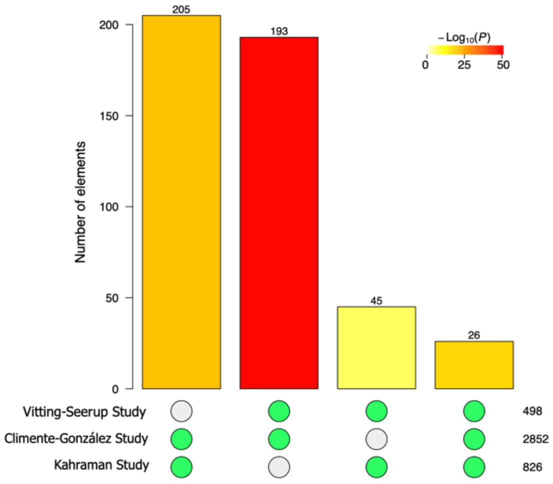

Liver Hepatocellular Carcinoma (LIHC)

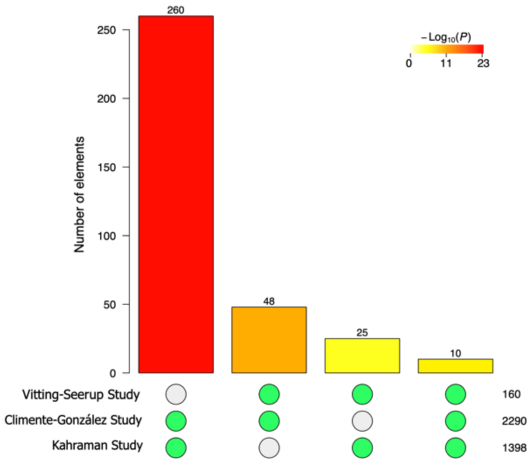

Lung Adenocarcinoma (LUAD)

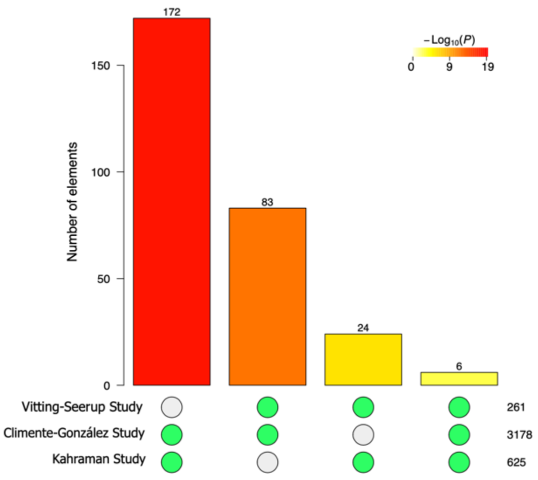

Lung Squamous Cell Carcinoma (LUSC)

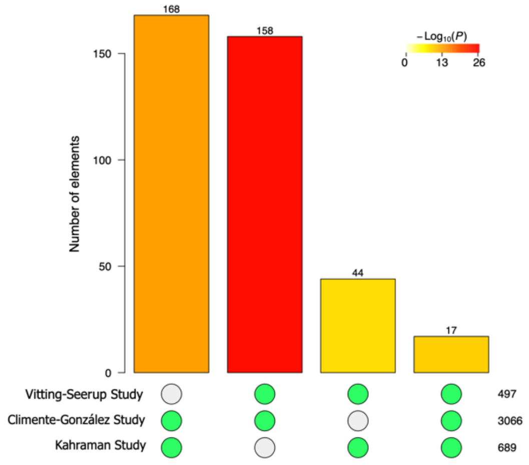

Prostate Adenocarcinoma (PRAD)

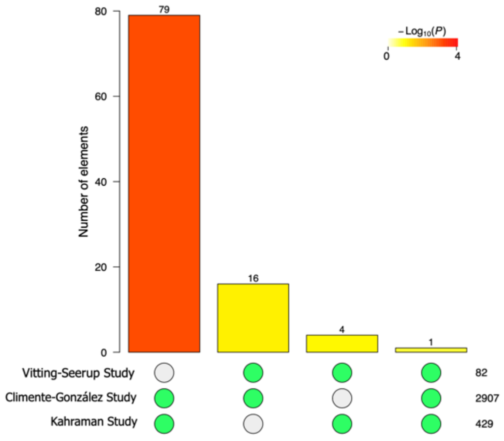

Thyroid Carcinoma (THCA)

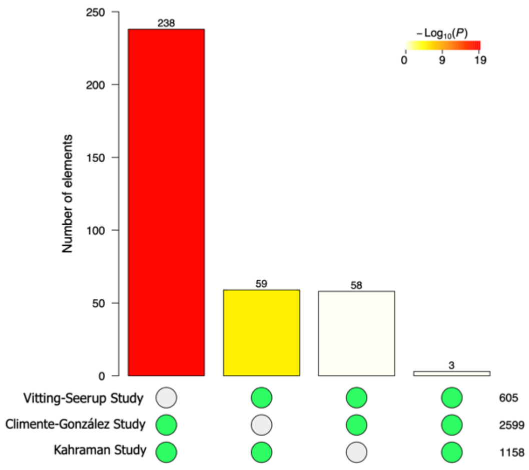

Kidney Renal Cell Carcinoma, Clear Cell and Papillary (KIRC + KIRP)

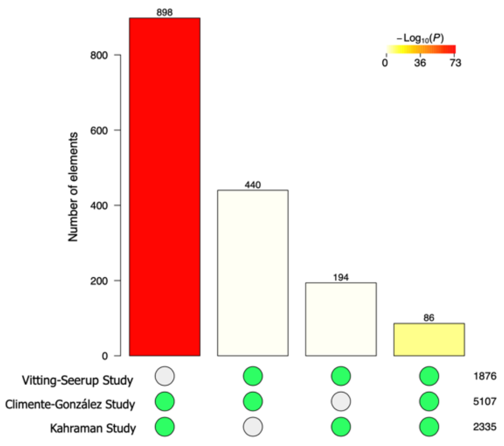

Supplement: Supplementary file 1 [file DataSheet2.pdf]
