# Supplementary material for: Probing Isoform Switching Events in Various Cancer Types: Lessons From Pan-Cancer Studies
Source: Front Mol Biosci. 2021 Nov 23;8:726902. doi: 10.3389/fmolb.2021.726902 (PMC8650491; doi:10.3389/fmolb.2021.726902)
Supplement: Supplementary file 2 [file DataSheet1.pdf]

Supplementary information

Probing Isoform Switching Events inVarious Cancer Types: Lessons From Pan-Cancer Studies

Tülay Karakulak, Holger Moch, Christian von Mering and Abdullah Kahraman

Gene Set Analysis

Breast Invasive Carcinoma (BRCA)

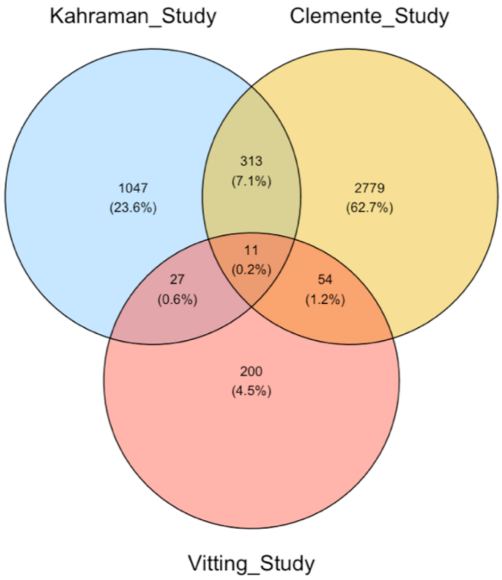

Genes Commonly Found in all 3 Studies:

TNC, ATXN3, FAM76B, CAP2, PNPLA7, PPAN, DACH1, CPLX1, SKA2, BICD2, HOXC6

Colon Adenocarcinoma (COAD)

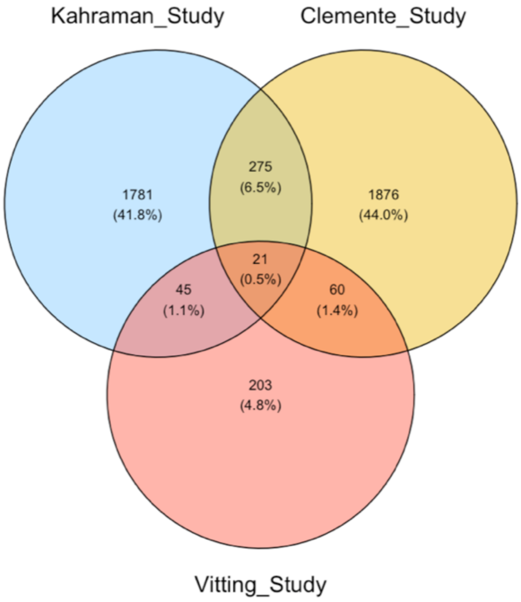

Genes Commonly Found in all 3 Studies:

C19orf60, OSBPL5, CD44, ZFYVE16, TLE2, ST6GALNAC1, LIMS2, CALD1, PNKD, SHC2, RIN2, MY O 10, C7orf50, AK3, RABGEF1, TSPAN7, FMNL3, ING2, PCCA, SH3BGR, BTN3A2

Kidney Chromophobe (KICH)

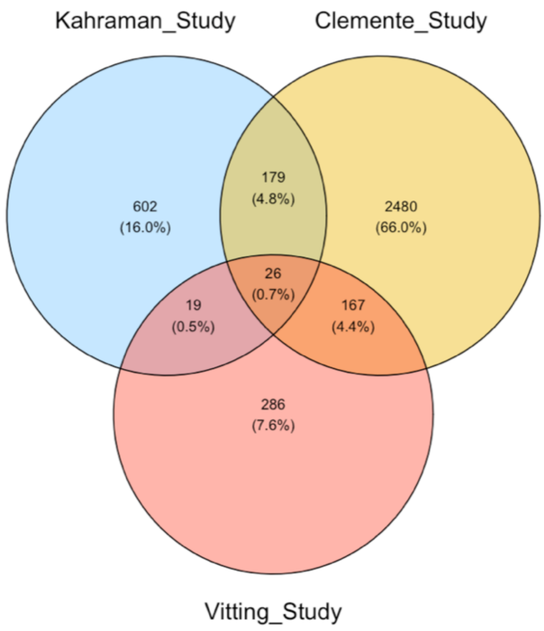

Genes Commonly Found in all all 3 Studies:

CD44, RC3H2, CEACAM1, NRCAM, CDADC1, GSPT1, CAV1, DNMBP, LIPA, NCOA7, KTN1, PDGFRA, BCAR3, RGS3, TARS2, ZNF385B, OBSCN, NAGS, BSCL2, PCMTD1, AP1S2, EXD3, ZNF44, WWP2, CTNND1, SCAMP5

Liver Hepatocellular Carcinoma (LIHC)

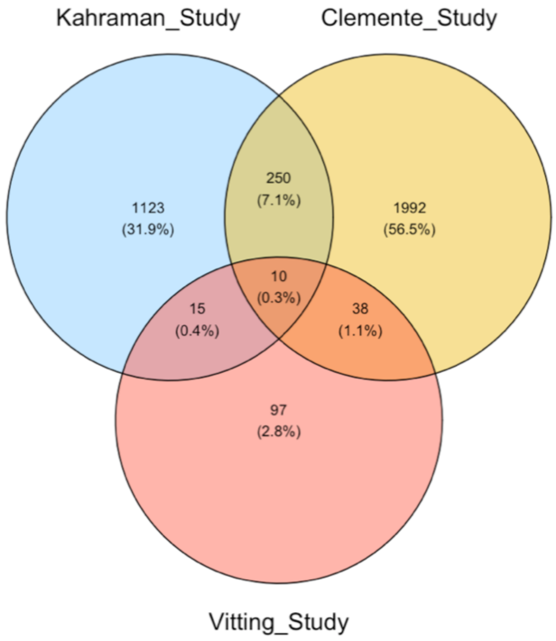

Genes Commonly Found in all 3 Studies:

KIF22, NXT2, NT5E, GBAS, PANK1, CXADR, MFAP4, NUDT6, FGGY, AQP1

Lung Adenocarcinoma (LUAD)

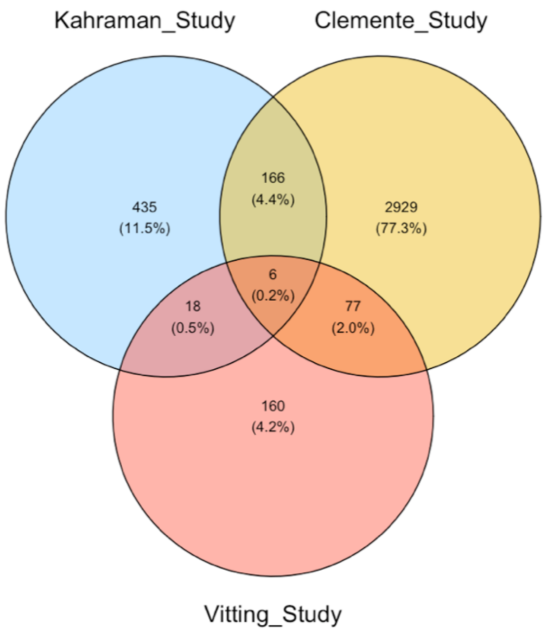

<u>Genes Commonly Found in all 3 Studies:</u>

SPAG9, LSR, ITM2C, CXADR, CPLX1, TNFRSF10C

Lung Squamous Cell Carcinoma (LUSC)

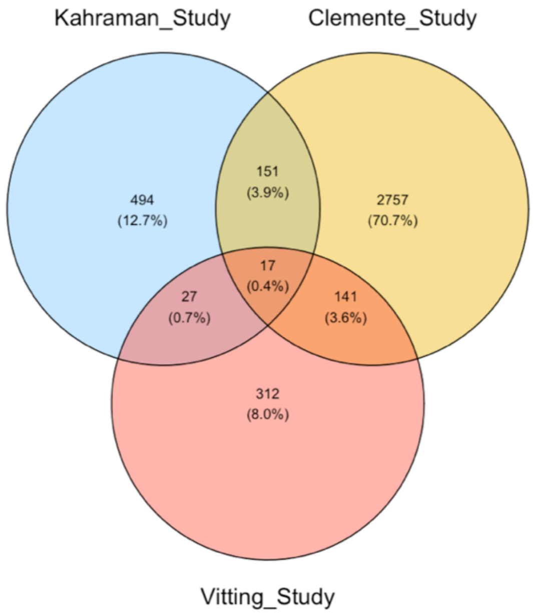

<u>Genes Commonly Found in all 3 Studies:</u>

CASP10, SDCCAG8, ATXN3, CECR1, DYRK1B, LSR, MEST, GAB1, CNOT2, MKLN1, SHROOM2, NTRK2, DST, IL17RE, PPFIBP2, WWOX, CTNND1

Prostate Adenocarcinoma (PRAD)

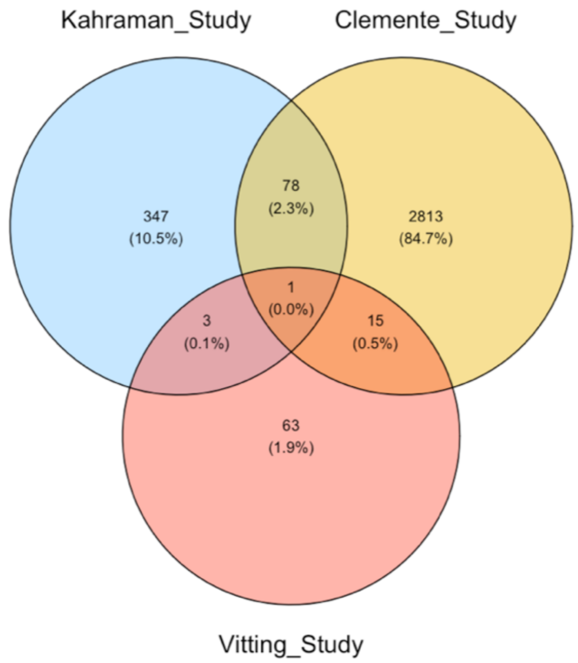

<u>Genes Commonly Found in all 3 Studies:</u>

VAMP1

Thyroid Carcinoma (THCA)

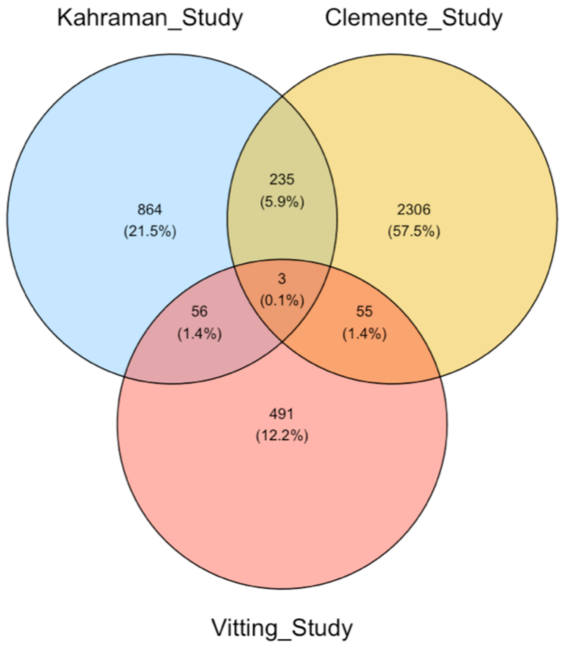

<u>Genes Commonly Found in all 3 Studies:</u>

CHF, Clorf198, FAM174B

Kidney Renal Cell Carcinoma, Clear Cell and Papillary (KIRC + KIRP)

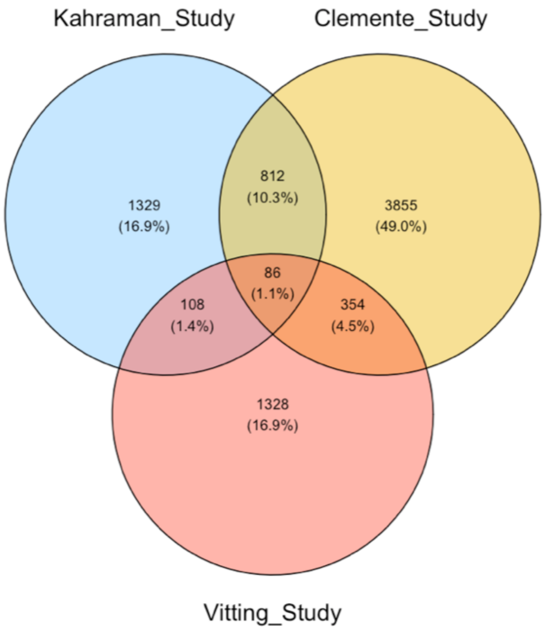

Genes Commonly Found in all 3 Studies:

C19orf60, MLXIPL, PLEKHH1, ATP2B4, SLC9A3R2, FGFR2, RASSF1, ATP2B1, BAZ2A, SMARCA2, EPS15, IPO11, MMP2, CECR1, MFNG, PATZ1, ASB9, FLT1, TAF1C, ILVBL, TIMM50, ADAP1, CAV1, DOCK8, DNMBP, LIPA, SHOC2, KLHL2, ST3GAL4, MVK, SCNN1A, BTN3A3, DOCK7, MXI1, ARAP3, ACOT9, IQSEC2, PSD4, EML2, TRPM4, EPS8L1, DIAPH1, DMGDH, PDGFRA, BIVM, ITM2C, CIB2, BIN1, FPGS, BCAR3, NCOA4, RNF185, SSBP2, DCDC2, ZNF185, TM7SF2, SCOC, ABI3BP, CXADR, RABGEF1, RMND1, AIFM1, SLC35B2, KALRN, MUM1, TBC1D24, STK36, HDAC11, MYO5B, TMCC1, GOLGA8A, EFCAB4A, GRAMD1C, LIMK2, SPNS3, AFMID, RBM33, FAM174B, MITF, ZNF559, TMEM201, ZNF44, WWP2, SCAMP5, PPME1, CUX1

Isoform Switch Analysis

Breast Invasive Carcinoma (BRCA)

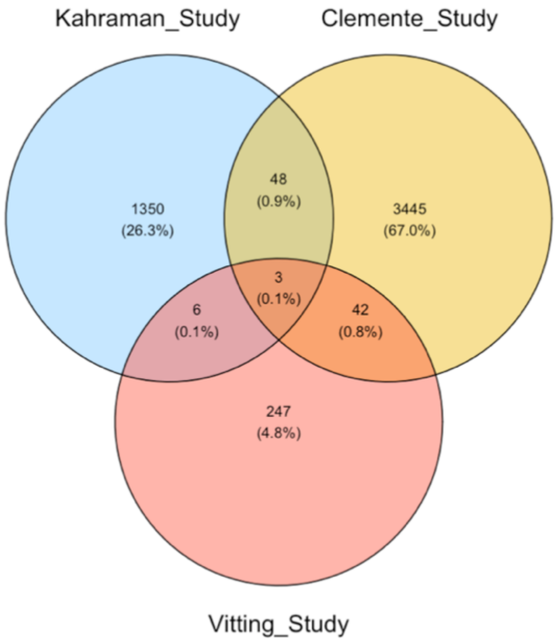

Isoform Switch Commonly Found in all 3 Studies:
Normal Isoform Cancer-specific Isoform (Gene Name)

uc003gbj_uc003gbi (CPLX1)
uc004aso_uc004asp (BICD2)
uc003ncb_uc011djb (CAP2)

Colon Adenocarcinoma (COAD)

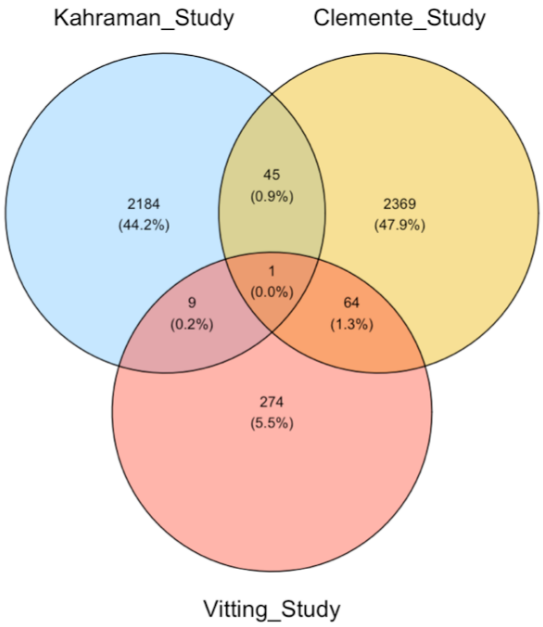

Isoform Switch Commonly Found in all 3 Studies:
Normal Isoform Cancer-specific Isoform (Gene Name)

uc002vhm_uc002vhq (PNKD)

Kidney Chromophobe (KICH)

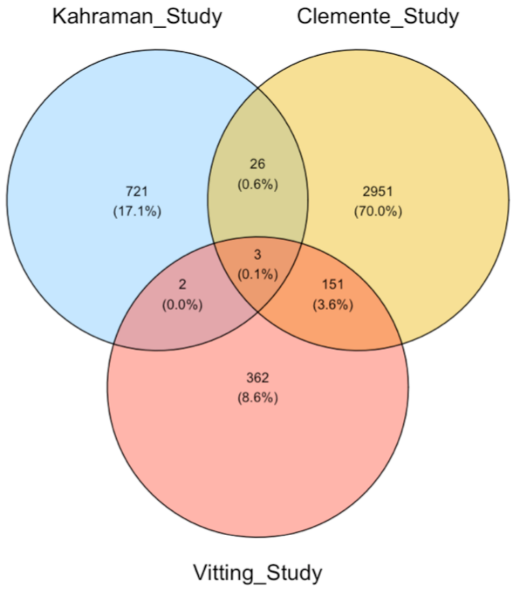

Isoform Switch Commonly Found in all 3 Studies:
Normal Isoform Cancer-specific Isoform (Gene Name)

uc002otv_uc002otw (CEACAM1)
uc001mvx_uc001mvw (CD44)
uc002unj_uc002unn (ZNF385B)

Liver Hepatocellular Carcinoma (LIHC)

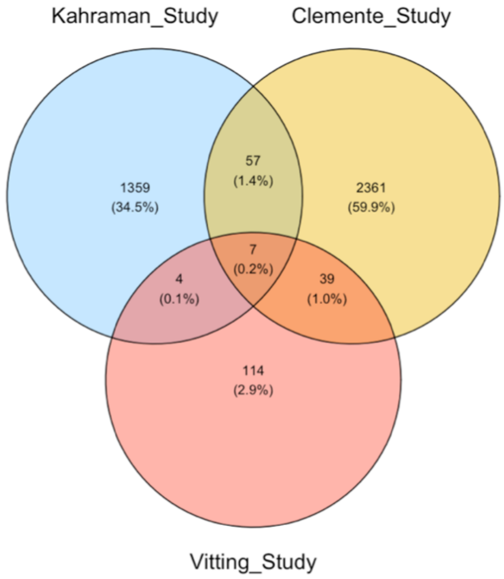

Isoform Switch Commonly Found in all 3 Studies:
Normal Isoform Cancer-specific Isoform (Gene Name)

uc003iew_uc003iex (NUDT6)
uc003tbv_uc010kwf (AQP1)
uc003pko_uc010kbr (NT5E)
uc003tre_uc003trf (NIPSNAP2)
uc002yki_uc002ykj (CXADR)
uc001kgn_uc001kgo (PANK1)
uc001qyu_uc001qyt (YBX3)

Lung Adenocarcinoma (LUAD)

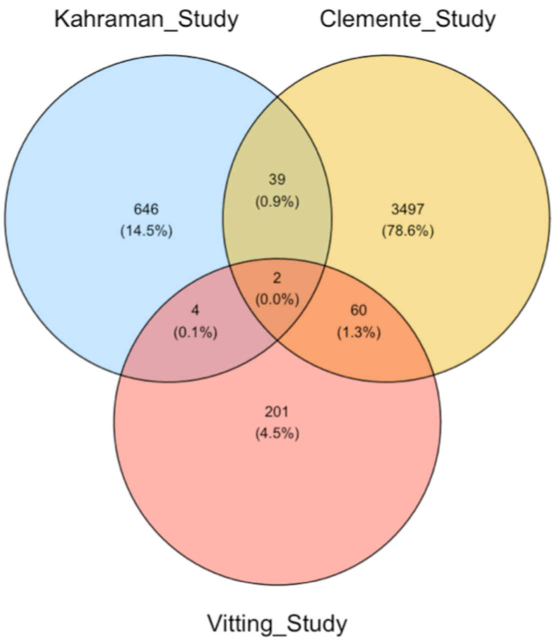

<u>Isoform Switch Commonly Found in all 3 Studies:</u>
<u>Normal Isoform Cancer-specific Isoform (Gene Name)</u>

uc003gbj_uc003gbi (CPLX1)
uc002yki_uc002ykj (CXADR)

Lung Squamous Cell Carcinoma (LUSC)

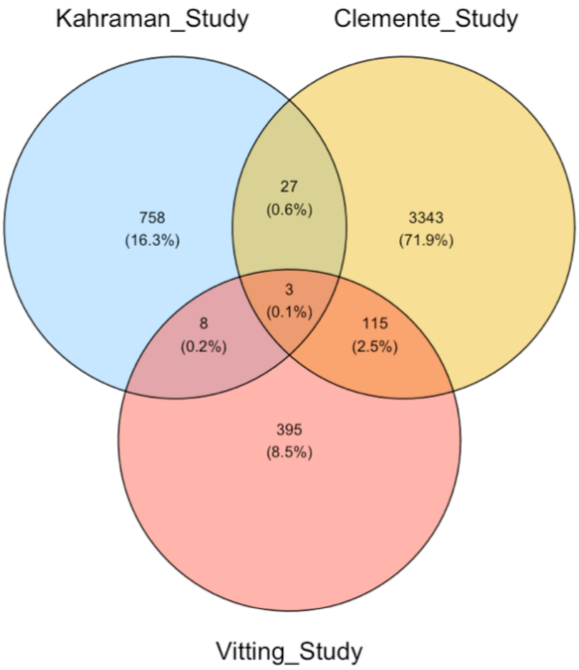

<u>Isoform Switch Commonly Found in all 3 Studies:</u>
<u>Normal Isoform Cancer-specific Isoform (Gene Name)</u>

uc002uxj_uc010ftb (CASP10)
uc002nym_uc002nyn (LSR)
uc003vqg_uc003vqc (MEST)

Prostate Adenocarcinoma (PRAD)

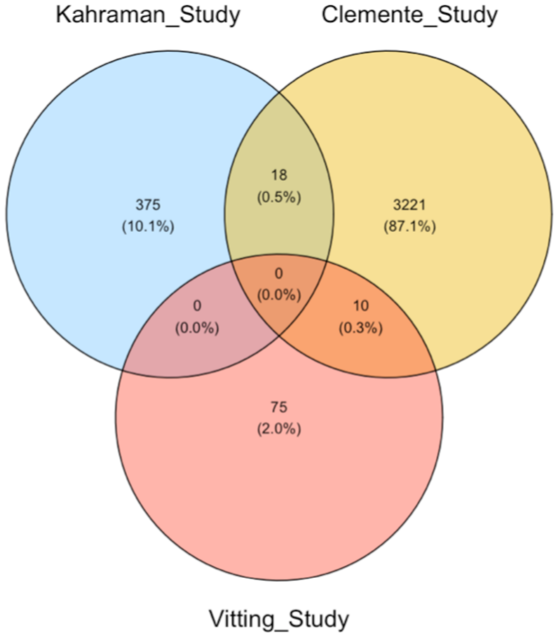

<u>Isoform Switch Commonly Found in all 3 Studies:</u>
<u>Normal Isoform Cancer-specific Isoform (Gene Name)</u>

No common switching event

Thyroid Carcinoma (THCA)

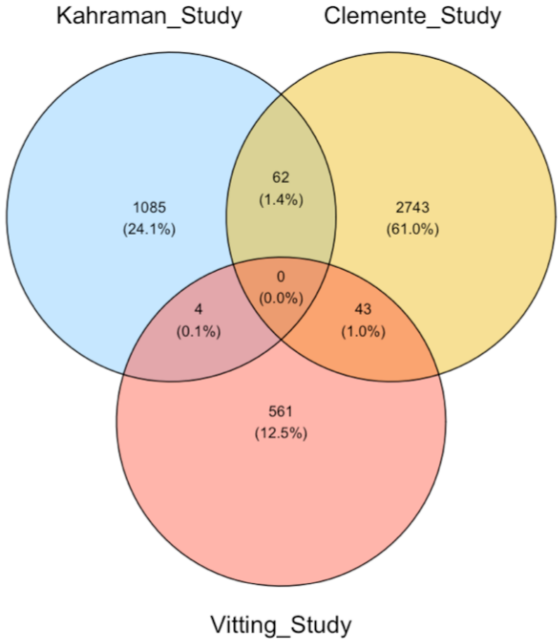

<u>Isoform Switch Commonly Found in all 3 Studies:</u>
<u>Normal Isoform Cancer-specific Isoform (Gene Name)</u>

No common switching event

Kidney Renal Cell Carcinoma, Clear Cell and Papillary (KIRC + KIRP)

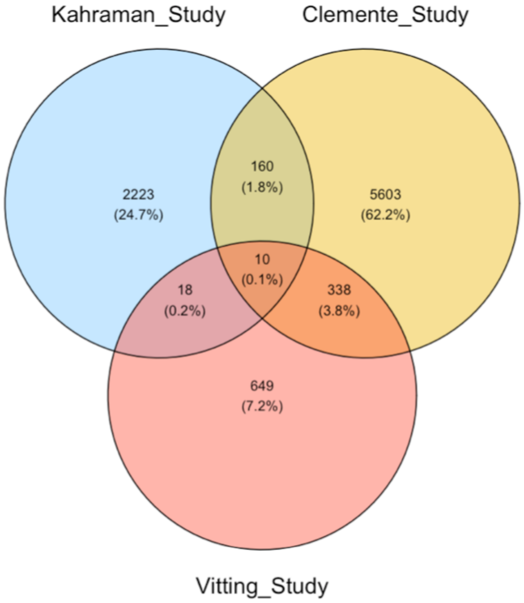

Isoform Switch Commonly Found in all 3 Studies:
Normal Isoform Cancer-specific Isoform (Gene Name)

uc001kza_uc001kyy (MXI1)
uc003akb_uc003ake (RNF185)
uc004evg_uc004evi (AIFM1)
uc002bdb_uc002bdc (CIB2)
uc002cok_uc002coi (SLC9A3R2)
uc001dpx_uc001dpy (BCAR3)
uc002yki_uc002ykj (CXADR)
uc004bsg_uc004bsi (FBGS)
uc003aks_uc003akr (PATZ1)
uc003eas_uc003eaq (GRAMD1C)

Normal Isoform Analysis

Breast Invasive Carcinoma (BRCA)

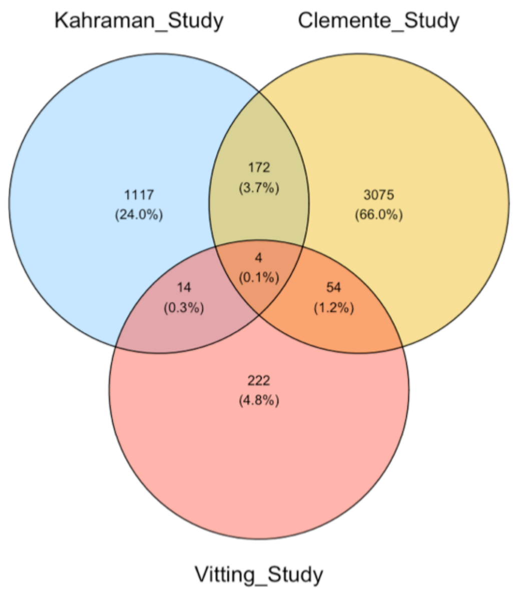

Normal Isoform Commonly Found in all 3 Studies: Normal Isoform

uc003gbj, uc004aso, uc003ncb, uc001sev

Colon Adenocarcinoma (COAD)

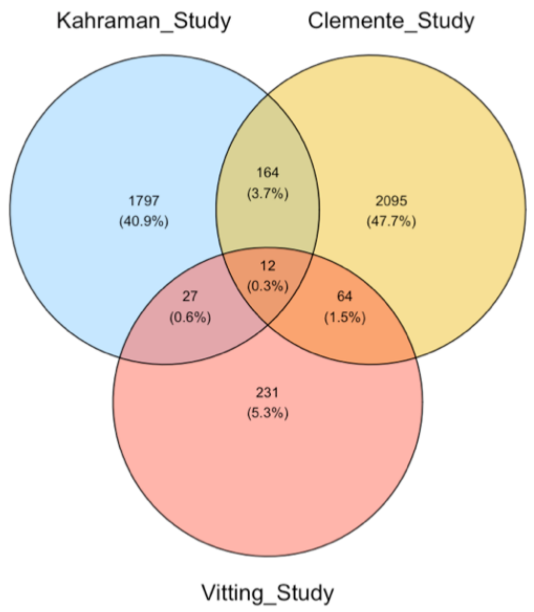

Normal Isoform Commonly Found in all 3 Studies: Normal Isoform

uc002tpa, uc001voo, uc001lxk, uc002njv, uc002vhm, uc002jsh, uc003kgq, uc002loq, uc003jft, uc003ziq, uc004deg, uc002yya

Kidney Chromophobe (KICH)

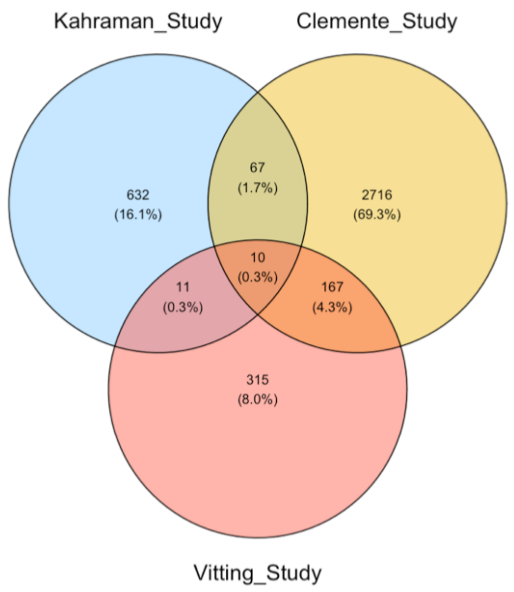

<u>Normal Isoform Commonly Found in all 3 Studies:</u>
<u>Normal Isoform</u>

uc002otv, uc001qba, uc004cxi, uc004cmp, uc001mvx, uc001nup, uc003vfc, uc003han, uc002unj, uc002ies

Liver Hepatocellular Carcinoma (LIHC)

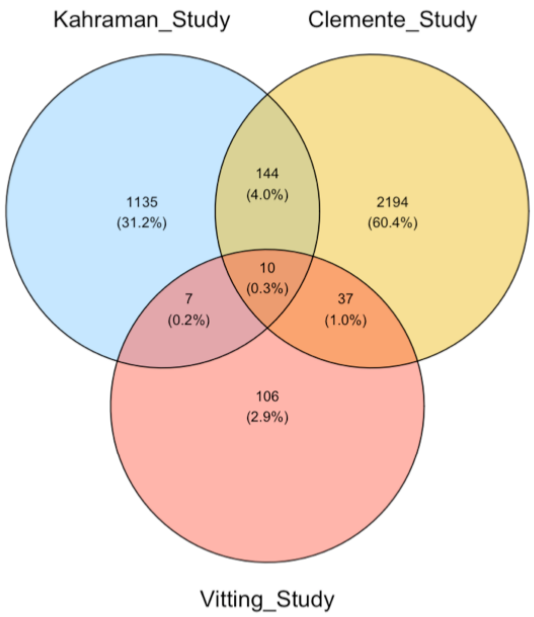

<u>Normal Isoform Commonly Found in all 3 Studies:</u>
<u>Normal Isoform</u>

uc003iew, uc003tbv, uc002gvt, uc003pko, uc003tre, uc002yki, uc001kgn, uc003hzc, uc001qyu, uc004eof

Lung Adenocarcinoma (LUAD)

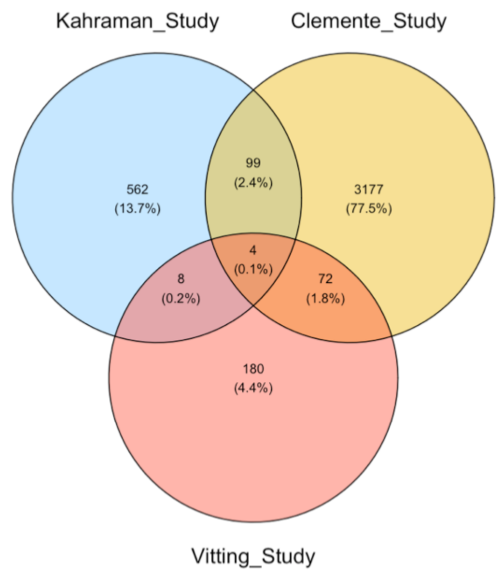

Normal Isoform Commonly Found in all 3 Studies:
Normal Isoform

uc002vqz. uc003gbj, uc003xcy, uc002yki

Lung Squamous Cell Carcinoma (LUSC)

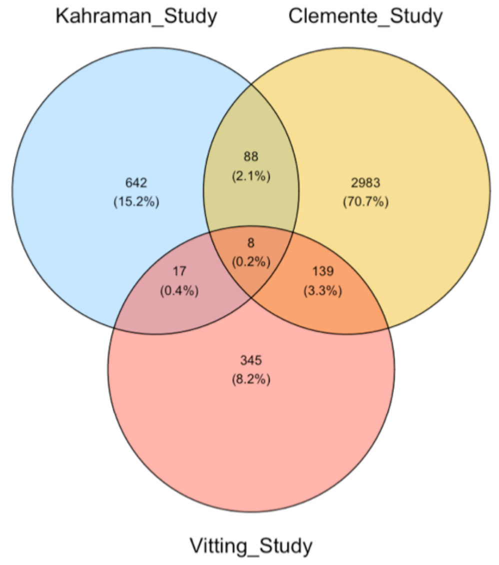

Normal Isoform Commonly Found in all 3 Studies:
Normal Isoform

uc002uxj, uc002nym, uc002ffk, uc004csu, uc011chq, uc009zro, uc003vqg, uc003btw

Prostate Adenocarcinoma (PRAD)

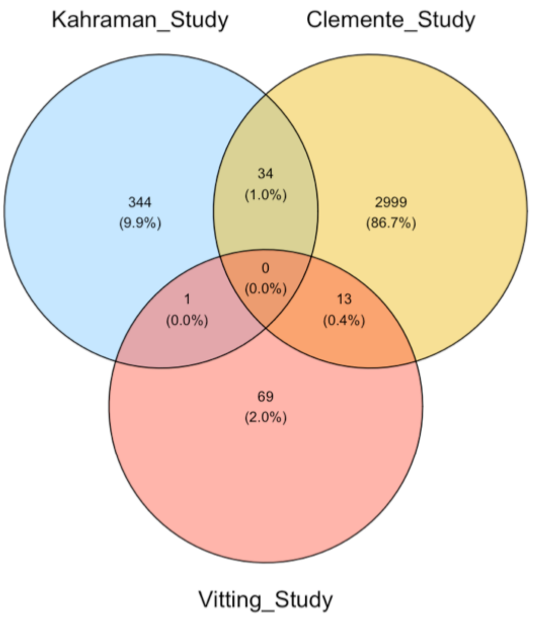

<u>Normal Isoform Commonly Found in all 3 Studies: Normal Isoform</u>

No common normal isoform

Thyroid Carcinoma (THCA)

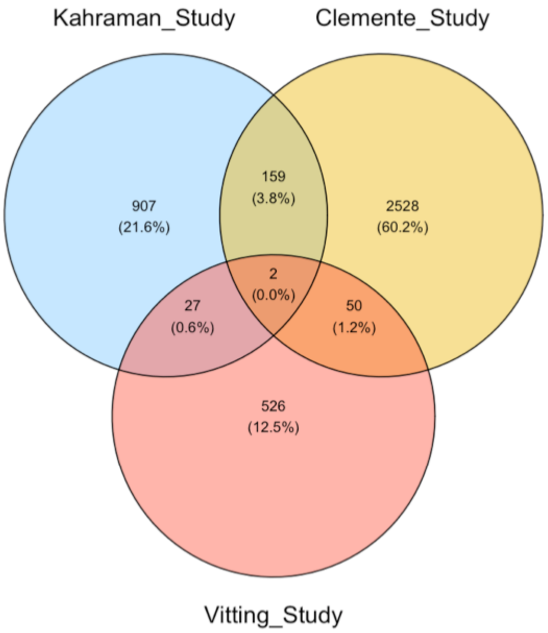

<u>Normal Isoform Commonly Found in all 3 Studies: Normal Isoform</u>

uc001hub, uc010boe

Kidney Renal Cell Carcinoma, Clear Cell and Papillary (KIRC + KIRP)

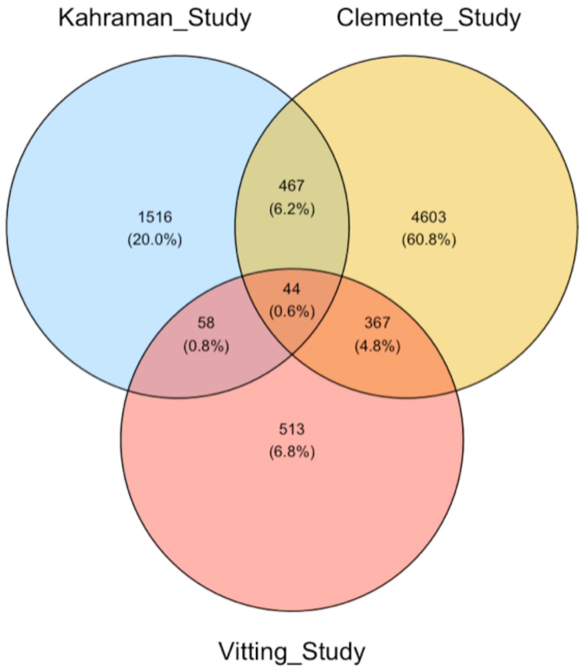

<u>Normal Isoform Commonly Found in all 3 Studies: Normal Isoform</u>

uc001csq, uc001kzl, uc001vps, uc002vqz, uc003oxd, uc002cqk, uc002njv, uc003dab, uc002bdb, uc003tyn, uc001xjl, uc003han, uc004cwk, uc004dap, uc003eas, uc001apy, uc003irb, uc009zvk, uc001kza, uc002pcn, uc002tob, uc003akb, uc001oct, uc004evg, uc010boe, uc003vcm, uc003sjo, uc001lrv, uc002zmj, uc002pmw, uc003ass, uc003llm, uc002cok, uc002nam, uc001dpx, uc002yki, uc004bsg, uc011che, uc002jva, uc003aks, uc002tjc, uc002qis, uc003kfs, uc003ndx

Cancer Isoform Analysis

Breast Invasive Carcinoma (BRCA)

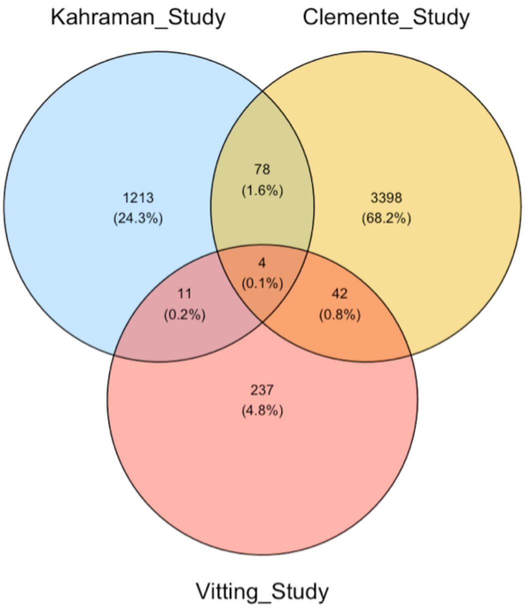

Cancer Isoform Commonly Found in all 3 Studies: Normal Isoform

uc003gbi, uc004asp, uc002uhz, uc011djb

Colon Adenocarcinoma (COAD)

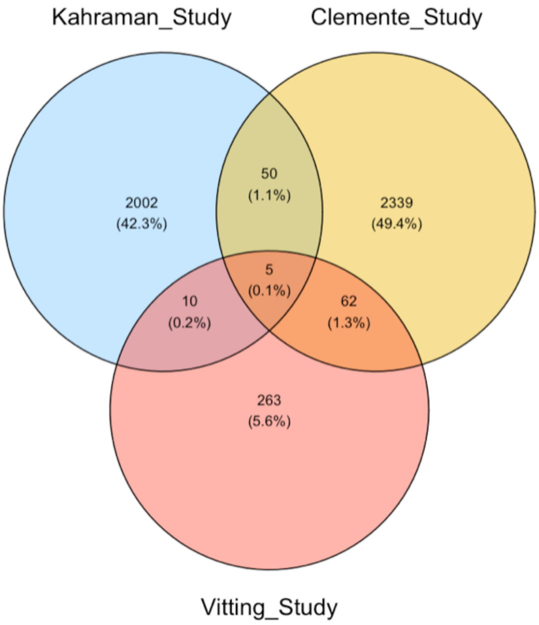

Cancer Isoform Commonly Found in all 3 Studies: Normal Isoform

uc003sju, uc003tvh, uc002uhz, uc002vhq, uc009xkg

Kidney Chromophobe (KICH)

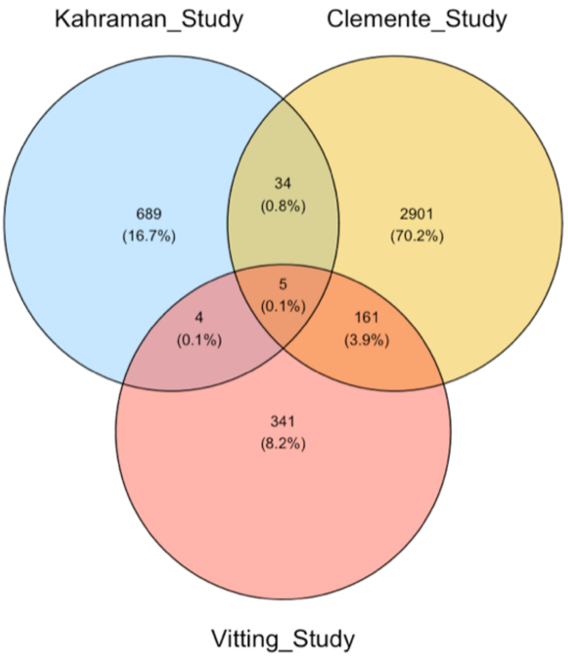

<u>Cancer Isoform Commonly Found in all 3 Studies: Normal Isoform</u>

uc002otw, uc003qai, uc001mvw, uc001dpy, uc002unn

Liver Hepatocellular Carcinoma (LIHC)

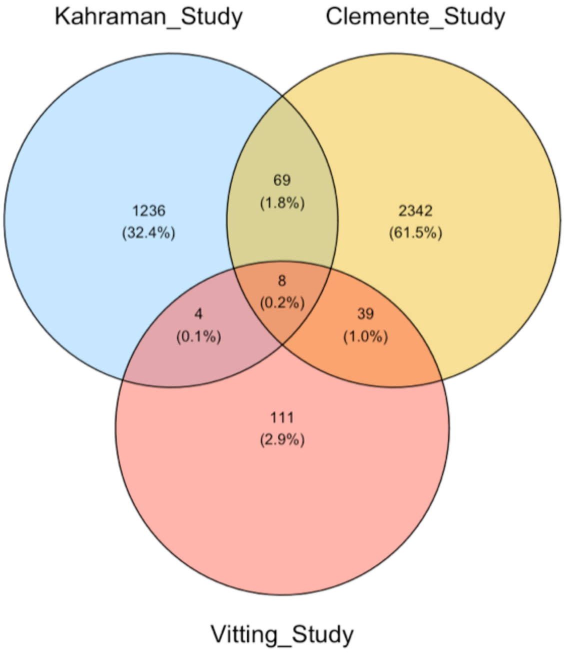

<u>Cancer Isoform Commonly Found in all 3 Studies: Normal Isoform</u>

uc003iex, uc010kwf, uc010kbr, uc003trf, uc002ykj, uc001kgo, uc001qyt, uc002dts

Lung Adenocarcinoma (LUAD)

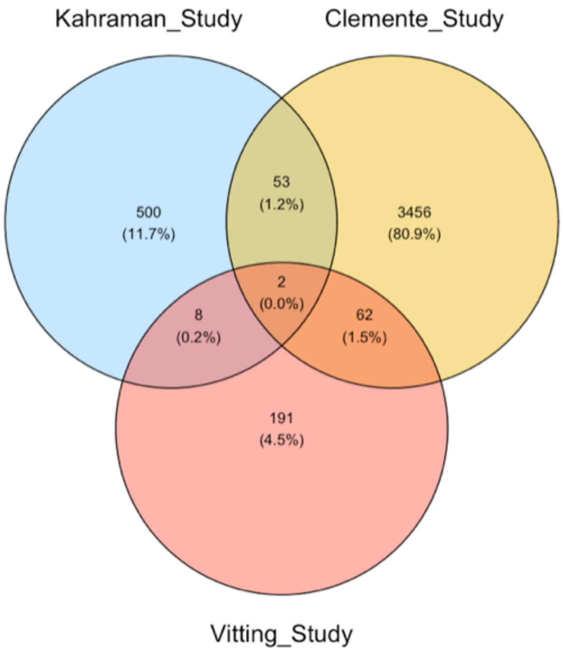

Cancer Isoform Commonly Found in all 3 Studies: Normal Isoform

uc003gbi, uc002ykj

Lung Squamous Cell Carcinoma (LUSC)

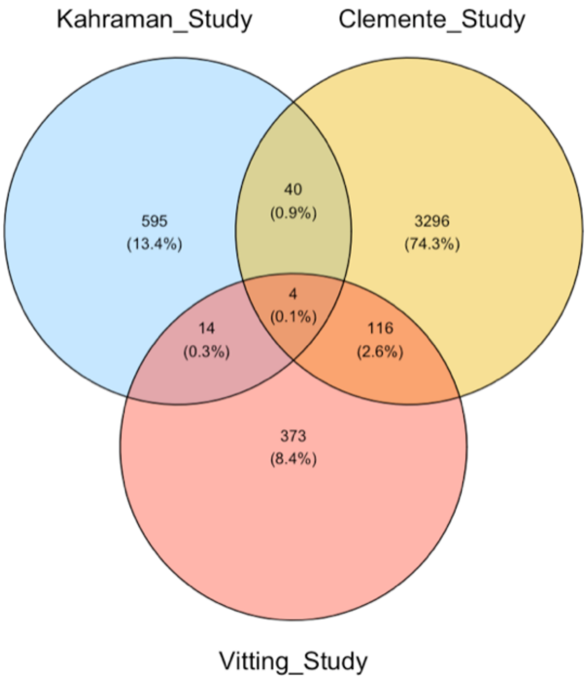

Cancer Isoform Commonly Found in all 3 Studies: Normal Isoform

uc010ftb, uc002nyn, uc003pdc, uc003vqc

Prostate Adenocarcinoma (PRAD)

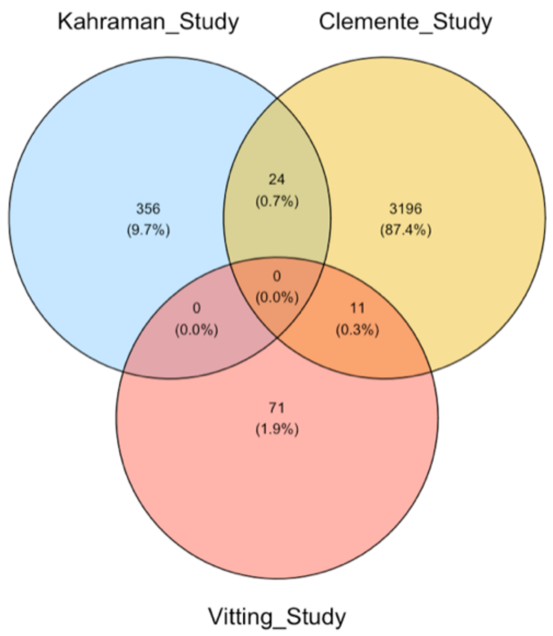

<u>Cancer Isoform Commonly Found in all 3 Studies: Normal Isoform</u>

No common cancer isoform

Thyroid Carcinoma (THCA)

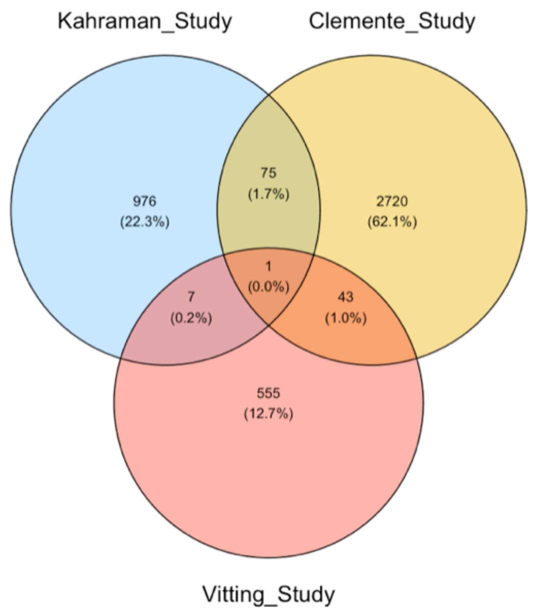

<u>Cancer Isoform Commonly Found in all 3 Studies: Normal Isoform</u>

`uc009xkg`

Kidney Renal Cell Carcinoma, Clear Cell and Papillary (KIRC + KIRP)

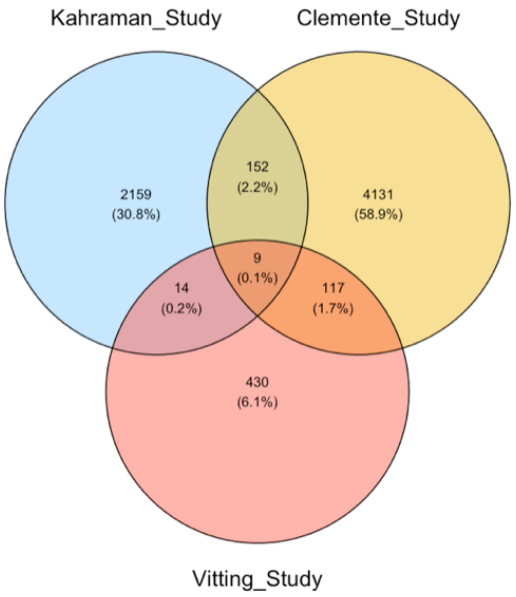

Cancer Isoform Commonly Found in all 3 Studies: Normal Isoform

uc001kyy, uc003ake, uc003tvh, uc003ehk, uc002bdc, uc001dpy, uc002ykj, uc003akr, uc003eaq
